# TNF-α-preconditioning enhances analgesic efficacy of mesenchymal stem cell-derived extracellular vesicle in neuropathic pain *via* miR-101b-3p targeting Nav1.6

**DOI:** 10.1016/j.bioactmat.2025.07.029

**Published:** 2025-07-26

**Authors:** Lanyu Zhang, Jinping Wang, Jin Liu, Juan Xin, Yuan Tan, Donghang Zhang, Tao Zhu, Cheng Zhou

**Affiliations:** a Department of Anesthesiology, West China Hospital, Sichuan University, Chengdu, 610041, China; b Research Center of Anesthesiology, National-Local Joint Engineering Research Centre of Translational Medicine of Anesthesiology, West China Hospital, Sichuan University, Chengdu, 610041, China

**Keywords:** Pain, Extracellular vesicle, MSC, Neuron, MicroRNA

## Abstract

Mesenchymal stem cell-derived extracellular vesicle (MSC-EV) has shown promise for pain relief, but its efficacy is limited. Preconditioning MSC with tumor necrosis factor-α (TNF-α) may enhance their therapeutic potential; however, the impact on analgesia and underlying mechanisms remains unclear. Here, we investigated the analgesic effects of EV from TNF-α-preconditioned MSC (T-EV) in a chronic constriction injury (CCI) mouse model and examined the molecular mechanisms involved. Following intrathecal injection, T-EV produced greater improvements in mechanical and thermal pain thresholds than control MSC-EV (C-EV), achieving enhanced pain relief for two weeks. Whole-cell patch-clamp recordings revealed that T-EV markedly decreased both the firing rate and action potential amplitude of dorsal root ganglion (DRG) neurons. RNA sequencing revealed that T-EV was enriched in miR-101b-3p. Silencing miR-101b-3p in T-EV abolished their enhanced analgesic effects and reversed DRG hyperexcitability. Moreover, miR-101b-3p was shown by luciferase assays to bind directly to the 3′UTR of Nav1.6, suppressing its expression. Engineered MSC-derived nanovesicle overexpressing miR-101b-3p replicated the increased pain relief observed with T-EV. These findings demonstrate that TNF-α preconditioning improves the analgesic potency of MSC-EV by delivering miR-101b-3p, which downregulates Nav1.6 and decreases DRG hyperexcitability. This study supports the therapeutic potential of miR-101b-3p-enriched vesicle as a novel strategy for treating neuropathic pain.

## Highlights

• T-EV exerts significantly stronger analgesic effects in the mouse model of neuropathic pain compared to the control EV.
• The enhanced analgesic efficacy of T-EV is mediated by increased concentration of miR-101b-3p within the vesicles.
• MiR-101b-3p directly targets and downregulates Nav1.6 expression, contributing to the potent analgesic response.
• Nanovesicle-encapsulated miR-101b-3p produces a similar analgesic effect to T-EV, highlighting its therapeutic potential.

## Graphical abstract

**Figure:**
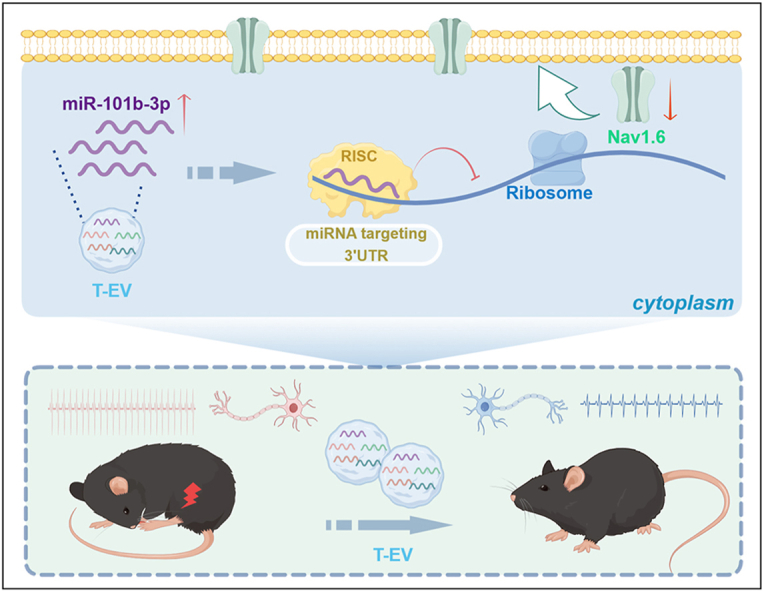

## 1 Introduction

Chronic neuropathic pain, which mainly results from injury, disease, or inflammation of the somatosensory system, is characterized by allodynia, hyperalgesia, or spontaneous pain [1]. This condition affects approximately 3 %–17 % of the global population, yet current treatment options demonstrate limited efficacy and suboptimal tolerability [[2], [3], [4]]. The clinical utility of first-line pharmacotherapies for neuropathic pain, including antiepileptics, opioid analgesics, and tricyclic antidepressants, is fundamentally constrained by a narrow therapeutic window. Dose-dependent adverse effects and tolerance development frequently preclude dose escalation to achieve sustained analgesic efficacy. Non-pharmacological alternatives, including neuromodulation therapies and ablative neurosurgical procedures, while mechanistically promising, present inherent procedural risks ranging from device-related infections to irreversible iatrogenic neural damage. Despite a marked increase in clinical studies on neuropathic pain over the past few decades, effective therapy remains a major challenge.

Regenerative medicine offers a promising therapeutic approach with significant translational potential, particularly in tissue repair and immune modulation [5,6]. In recent years, this field has been increasingly applied to chronic pain management, evolving into what is now termed “regenerative pain medicine” [[7], [8], [9]]. Clinical studies have demonstrated that administering mesenchymal stem cell (MSC) can alleviate chronic pain in patients [10,11]. Extracellular vesicle (EV), a key mediator of the paracrine signaling mechanisms of MSC, has emerged as a promising alternative to cell-based therapies, owing to its nonimmunogenic properties [12]. MSC-derived EV (MSC-EV) exhibits moderate analgesic effects on neuropathic pain models [[13], [14], [15]]. However, they do not fully restore the pain threshold, and their efficacy in treating neuropathic pain remains limited [13,14]. Furthermore, the precise mechanisms underlying the analgesic effects of MSC-EV are not yet fully understood. Most previous studies attribute the analgesic effect of MSC-EV to the neuroimmune modulation ability [13,14]. However, the above views do not fully encompass the complexity of their analgesic mechanisms in neuronal sensitization of neuropathic pain [16,17], especially what is the critical molecular regulator of MSC-EV in peripheral nerve injury and/or neural inflammation. Therefore, direct evidence that supports the regulation of neurons by MSC-EV in neuropathic pain remains scarce. Current neuropathic pain management faces challenges, however, MSC-EV uniquely overcomes these limitations by delivering multimodal therapeutics that concurrently address neuroinflammation, repair neural damage, and maintain analgesic effectiveness without developing tolerance.

MSC possess functional plasticity, enabling their therapeutic properties to be modulated by cytokines, growth factors, and environmental cues like hypoxia. After transplantation, MSC sense and respond to microenvironment signals, a process called “MSC licensing” [18]. The microenvironment plays a crucial role in modulating the therapeutic potential of MSC by influencing the activation or suppression of MSC gene expression [19,20]. Small-molecule pretreatment has been explored as an important strategy to increase the therapeutic potential of MSC-EV. Among these small molecules, tumor necrosis factor-alpha (TNF-α) has been identified as an effective molecule that enhances the therapeutic effects of MSC-EV on retinal ischemia-reperfusion injury, periodontal bone loss, and wound healing [[21], [22], [23]]. Although TNF is a proinflammatory factor produced under various injury conditions, it can reprogram and enhance the functional properties of MSC. The Fas/Fap-1/Cav-1 axis in MSC regulates the secretion of interleukin-1 receptor antagonist (IL-1RA) within EV. TNF-α critically enhances Fas and Fap-1 expression in MSC, thereby promoting IL-1RA release and enhancing the therapeutic effect on wound healing [22]. TNF-α-stimulated gingival MSC-derived EV demonstrated enhanced neuroprotective effects in a retinal ischemia-reperfusion injury model, which was attributed to the enrichment of miR-21–5p [23]. However, whether TNF-α-stimulated MSC-EV produces enhanced analgesic effects on neuropathic pain is still unknown. Theoretically, TNF-α is an important cytokine within the microenvironment of chronic pain in both the central and peripheral nervous systems, where it upregulates the expression of ion channels, thereby promoting neuronal sensitization [24,25]. Given these findings, it is essential to investigate whether TNF-α alters the molecular composition of MSC-EV, and how this alteration may influence ion channel expressions in neuronal sensitization of chronic pain. This study hypothesized that TNF-α enhances the therapeutic potential of MSC-EV in pain management by modulating their molecular composition, thereby regulating ion channel expression in neurons during neuropathic pain.

In this study, the results demonstrated that early intervention with EV derived from TNF-α-primed MSC (T-EV) led to superior analgesic effects compared with those of EV derived from common MSC. T-EV effectively suppresses excessive neuronal excitability after nerve injury. Through RNA sequencing and subsequent validation, miR-101b-3p was identified as a key mediator contributing to the enhanced analgesic effects of T-EV. Furthermore, miR-101b-3p expression has been reported to be decreased in patients with neuropathic pain and is closely associated with pain regulation [26]. Further analysis revealed that miR-101b-3p, which was enriched in T-EV, selectively targeted Nav1.6 in dorsal root ganglion (DRG) neurons to suppress neuronal excitability. As a key voltage-gated sodium channel isoform, Nav1.6 is critically involved in neuronal hyperexcitability and pain sensitization, making it a promising target for neuropathic pain intervention [27]. Therefore, this study demonstrated the enhanced analgesic potential of EV derived from TNF-α-activated MSC, elucidating their direct impact on neuronal excitability and the underlying mechanisms mediating pain relief in neuropathic pain. Finally, given its greater accessibility and yield than EV does, nanovesicle (NV) offers a practical alternative for loading miR-101b-3p, achieving the same analgesic effect as T-EV. These findings could lead to a promising translation for clinical management.

## 2 Methods

### 2.1 Cell culture

#### 2.1.1 Primary MSC culture and pretreatment

Different batches of primary mouse bone marrow-derived mesenchymal stem cells from 3- to 4-week-old C57BL/6J mice were obtained from Cyagen Biosciences Inc. (catalog number MUBMX-01001, China) [28]. The cells were cultured in Dulbecco's Modified Eagle's Medium/Nutrient Mixture F-12 (DMEM/F12) (Gibco™, USA) supplemented with 10 % fetal bovine serum (FBS, BA1650-175, BIOEXPLORER, China) and 1 % penicillin-streptomycin (Gibco™, USA) under standard conditions in a humidified atmosphere containing 5 % CO_2_. Primary human bone marrow-derived MSC was obtained from Cyagen Biosciences Inc. (catalog number HUXMA-01001, China). The hMSC was cultured in α-Minimum Essential Medium (α-MEM, GibcoTM, USA) supplemented with 10 % FBS and 1 % penicillin-streptomycin under standard conditions in a humidified atmosphere containing 5 % CO_2_. Upon the MSC reaching 90 % confluence, the cell was passaged. The culture medium was subsequently replaced with fresh serum-free DMEM/F12 or α-MEM medium after washing twice with PBS, and the cells were treated with either 50 ng/ml TNF-α or an equal volume of PBS for 48 h. The conditioned medium was collected at the end of the treatment period for further analysis. For virus transfection, VSVG-LENTAI-hU6-shRNA (miR-101b-3p)-esEF1A-MataGFP-IRES-PuroR-WPRE-pA or VSVG-LENTAI-hU6-shRNA (NC)-esEF1A-MataGFP-IRES-PuroR-WPRE-pA was obtained from Taitool Bioscience Inc. and transfected with 50 μg/ml polybrene and 1 % puromycin into MSC for 12 h, after which the medium was changed to normal culture medium supplemented with 0.2 % puromycin. The following culture process was performed as described above. The sequences used to knock down miR-101b-3p in MSC were as follows:

shRNA-miR-101b-3p: ACCGAGCTATCACAGTACTGTACTTTTTTT

shRNA-NC: CTCGATGGAAAATACTCCGAG.

#### 2.1.2 Cell line culture

The neuroblastoma-dorsal root ganglion hybrid 7/23 (ND7/23, SCSP-5026, Cell Bank of the Chinese Academy of Sciences, China) hybrid cell line and the human embryonic kidney 293t (HEK293t, Shanghai Jinyuan Biotechnology Co., China) cell line was cultured in DMEM supplemented with 10 % FBS and 1 % penicillin-streptomycin. Cultures were incubated under standard conditions in a humidified atmosphere containing 5 % CO_2_.

#### 2.1.3 Primary DRG neuron culture

The extraction method for the primary neurons was modified and performed as previously reported [29]. The right lumbar L4-5 DRGs from the 8-week-old C57BL/6J mice were collected in a 35-mm tissue culture dish and digested in 2.5 mg/ml collagenase (BioFroxx, German) for 30 min at 37 °C, followed by the addition of 1.25 % trypsin (Gibco™) for another 20–30 min. Then, a complete medium was added to neutralize the trypsin. Neurons were seeded on laminin-coated (Sigma-Aldrich, USA) coverslips in 35-mm tissue culture dishes (Corning, USA) and maintained at 37 °C with neurobasal medium (Gibco™, USA) supplemented with 2 % B27 (Gibco™, USA), 1 % glutamine (Gibco™, USA), and 1 % penicillin and streptomycin in an atmosphere of 5 % CO_2_.

### 2.2 EV extraction and identification

The EV in the supernatant from MSC was isolated *via* an optimized differential centrifugation method, as previously described [30,31]. The supernatant was subsequently centrifuged at 300×*g* for 5 min to remove the cell pellet, after which it was centrifuged at 2000×*g* for 20 min to discard the cell debris. Then the supernatant was centrifuged at 10,000×*g* for 30 min to remove the large vesicle by a high-speed centrifuge (Beckman, Avanti J-26S XP, USA) in a special centrifuge tube (Beckman, #356011, USA). Finally, the medium was ultracentrifuged at 120,000×*g* for 120 min by a super-speed centrifuge (Beckman, Optima XPN-100, rotor 42Ti/32Ti, USA) in a special centrifuge tube (Beckman, #355622/#344058, USA). The sediment consisted of the small EV and was washed with PBS for another round of ultracentrifugation. EV was resuspended in 100–150 μl PBS, and the microprotein BCA assay (#23227, Thermo Fisher Scientific, USA) was used to test the protein concentration of the vesicle. Then the EV was stored at −80 °C. Fresh EV was also verified *via* transmission electron microscopy (TEM, JEOL, Japan), nanoparticle tracking analysis (NTA, ZETAVIEW, Germany), and Western blotting [31].

### 2.3 Transmission electron microscope

EV was diluted in cold PBS and applied to an electron microscopy grid for 5 min at room temperature. Following this, the EV was negatively stained with 0.75 % uranyl acetate dihydrate for another 5 min. The grids were washed three times with PBS, and excess liquid was gently removed using absorbent paper. Finally, the samples were examined under a transmission electron microscope (JEM-1400FLASH, JEOL, Japan) to assess the morphology of the EV.

### 2.4 Animals

C57BL/6J male mice were housed in a specific pathogen-free (SPF) condition. The condition was a temperature-controlled (22–24 °C) and humidity-controlled (40 %–60 %) room with a 12-h light (7:00–19:00)/12-h dark cycle and had ad libitum access to food and water. The Animal Ethics Committee approved the study protocol, and it was conducted following the Animal Research Reporting *In Vivo* Experiments (ARRIVE) guidelines.

### 2.5 Chronic constriction injury (CCI) model

Seven-week-old C57BL/6 wild-type mice, under 2 % isoflurane inhalation anesthesia, were placed in the right lateral recumbent position as previously reported [32]. The surgical procedure was performed on the right side, with the region between the greater trochanter of the femur and the ischial tuberosity prepared and disinfected. After the area was draped, a skin incision was made. Blunt dissection of the muscles was carried out in layers to expose the main trunk of the sciatic nerve. The sciatic nerve was then ligated *via* 4-0 silk sutures, with three separate ligatures placed approximately 1 mm apart. Following ligation, the incision was closed in layers. In the control group, the main trunk of the sciatic nerve was exposed without ligation.

### 2.6 Intrathecal injection

The intrathecal injection was performed as previously reported [33,34]. The mice were first anesthetized *via* inhalation of 2 % isoflurane and then positioned prone with the abdomen elevated to fully expose the intervertebral space after hair removal. A 30-G needle connected to a 25 μl Hamilton syringe was carefully inserted into the subarachnoid space between L5 and L6. A 10 μl volume of sterile solution was then slowly injected after a sudden tail flick was observed, indicating appropriate needle placement. The composition of the injected sterile solutions differed among experimental groups and included: (1) phosphate-buffered saline (PBS) and EV; (2) agomir and its negative control (NC); or (3) nanovesicles (NV). The needle was left in place for 2 min to ensure proper delivery of the solution. Following this, the needle was withdrawn, and the mouse was allowed to awaken.

### 2.7 *In vivo* tracing of DiR-labeled vesicle

EV or NV was labeled with DiR (#HY-D1048, MedChemExpress, USA) and then pelleted by ultracentrifugation at 120,000×*g* for 120 min. Following washing with PBS, DiR-labeled EV or NV was resuspended in PBS. The labeled EV or NV was administered intrathecally to mice on Day 1. Mice were imaged using an In Vivo Imaging System (IVIS, PerkinElmer, USA) on Day 1 and at subsequent time points until the signal was no longer detectable. Maximum radiant efficiency was quantified using the IVIS software. The maximal diffusion distance of DiR-labeled EV and NV from the injection site was determined on the second day post-injection.

### 2.8 PKH26 labeling and tracking of EV

EV was labeled using PKH-26 (Sigma-Aldrich, USA). The EV pellet was first resuspended in 1 ml of Diluent C. 4 μL of PKH-26 stock solution was added to 1 ml of Diluent C and mixed thoroughly to label the EV. This PKH-26 solution was then incubated with the EV suspension for 5 min. To terminate the staining process, 200 μL of FBS was added to the mixture to neutralize the dye. The labeled EV was then subjected to ultracentrifugation at 120,000×*g* for 120 min at 4 °C. Following centrifugation, the pellet containing PKH-26-labeled EV was washed with PBS and centrifuged again to remove excess dye. The final EV pellet was resuspended in PBS for subsequent experiments. For *in vivo* delivery, PBS or PKH26-EV (10 μl) was administered intrathecally to anesthetized mice. After 24 h, spinal cord and DRG tissues were harvested, cryosectioned, and imaged by confocal microscopy to quantify cellular EV internalization. For *in vitro* EV uptake assessment, primary DRG neurons were incubated with PKH26-labeled EV (5 μg/ml) for 48 h.

### 2.9 Behavioral tests

#### 2.9.1 Von-frey test

Before testing, the mice were acclimatized to the testing environment by being placed on a metal shelf for 1 h daily over 5 days. On the test day, the mice were individually placed in plexiglas chambers (8.0 × 8.0 × 8.0 cm) for 30 min to allow them to settle. The mechanical withdrawal threshold was assessed *via* a series of von-Frey filaments (0.008, 0.02, 0.04, 0.07, 0.16, 0.4, 0.6, and 1 g). Each filament was applied sequentially to the plantar surface of the right hind paw, and the absolute withdrawal threshold was determined *via* the up-down method as described in previous studies [35,36]. The final filament value was expressed as paw withdrawal threshold (PWT) for comparative analysis across the groups.

#### 2.9.2 Brush test

The brush test was conducted as previously described [37]. An artist's paintbrush was gently applied to the plantar surface of the right hindlimb. The brushing was repeated five times, with a 10-s interval between each application. After a 5-min rest period, the procedure was repeated once more. The mice's responses to the stimulus were recorded, and the percentage of responsiveness was calculated based on the number of responses observed.

#### 2.9.3 Pinprick test

The pinprick test was performed as previously described [37]. The mice were placed in a testing box similar to that used for the von-Frey test. A pin, attached to a 1 g calibrated von-Frey filament, was applied to the plantar surface of the right hind paw. The response time was recorded *via* a stopwatch.

#### 2.9.4 Hargreaves test

The mice were acclimated for 30 min in a testing chamber on an elevated transparent glass platform. After acclimation, the plantar surface of the hind paw was exposed to a radiant heat source (#37370, Ugo Basile, Varese, Italy) through transparent glass. The radiant heat was shut off when hind paw movement occurred or after 20 s to prevent tissue damage. Each thermal stimulus was repeated three times with a 10-min interval between trials. The mean latency to respond was calculated to assess thermal hyperalgesia.

#### 2.9.5 Cold pain test

Noxious cold thresholds were assessed *via* the dry ice assay, as previously described [38]. The mice were placed on a glass platform (5 mm) within a test box. Small pieces of dry ice were loaded into a 5 ml syringe, which was then positioned beneath the glass platform, directly under the hind paws. The latency for the mouse to withdraw its paw from the cold stimulus was recorded. Each hind paw was measured three times, and the average of these measurements was used for statistical analysis.

#### 2.9.6 Gait analysis

The VisuGait system (XR-FP101; Xinruan Information Technology, China) was used to analyze detailed gait parameters according to the manufacturer's protocol. The gait of all mice that successfully ran uninterrupted was recorded. The standing time, mean print area, and mean print intensity of ipsilesional and contralesional hindlimbs were analyzed by VisuGait software. Data were analyzed using the integrated VisuGait software, with results expressed as the ipsilesional/contralesional ratio for intergroup comparisons.

### 2.10 Enzyme-linked immunosorbent assay (ELISA)

The right L4 and L5 DRG tissues from 3 to 4 mouse models in each group were harvested on day 6 following CCI surgery. Total protein was extracted from DRG tissues by homogenizing the samples in PBS containing protease inhibitors (fresh weight to PBS ratio of 1:9) *via* magnetic beads. The homogenates were centrifuged at 5000 g for 10 min at 4 °C, and the supernatants were collected for further analysis. The levels of inflammatory mediators, including TNF-α, IL-1β, IL-6, IL-10, and CGRP, were quantified *via* commercially available ELISA kits (Ruixinbio, Quanzhou, China) according to the manufacturer's instructions.

### 2.11 Immunofluorescence staining

The mice were deeply anesthetized with isoflurane, followed by sequential perfusion with cold (4 °C) saline and 4 % paraformaldehyde transcardially. The DRG tissues from 3 to 4 mouse models in each group were carefully harvested on day 6 and subsequently fixed in 4 % paraformaldehyde for 24 h. After fixation, the tissues were subjected to a graded sucrose immersion process to cryoprotect the samples and then sectioned using a cryostat to obtain frozen sections. The frozen sections were washed three times with PBS and incubated sequentially with Triton X-100 to permeabilize the tissue and with goat serum to block nonspecific binding. Following blocking, sections were incubated overnight at 4 °C with the primary antibody (details in Supplementary Methods and Supplement Table 1). The next day, the sections were incubated with a fluorescently labeled secondary antibody at 37 °C for 2 h. After a final wash, the sections were mounted with DAPI-containing medium to stain the cell nuclei. Fluorescence images were captured *via* a confocal microscope (Nikon, A1RMP+, Japan). Fiji software analyzed the mean fluorescence intensity of images (National Institutes of Health, Bethesda, MD, USA).

### 2.12 Electrical transfection

The cells were resuspended in 1 ml of DMEM/F12. The cell suspension was extruded to obtain the nanovesicle (NV) by a MiniExtruder (Avanti® Polar lipids Inc) with a filter membrane according to the protocol reported previously [39]. The extruded mixture was filtered through a 0.22 μm filter to remove large impurities. The mixture was subsequently subjected to ultracentrifugation at 120,000×*g* for 2 h *via* a Beckman ultracentrifuge. The NV was then diluted in 100 μl of OptiMEM. The miR-101b-3p was added to the diluted NV solution to achieve a final concentration of 10^4 nM, and the mixture was transferred to an electroporation cup. Electroporation was performed using an electroporator (NEPA21, Japan) at 150 V for 5 ms, followed by transfer pulses at 20 V for 50 ms, as previously described [40]. After electroporation, the mixture was centrifuged again at 120,000×*g* for 90 min. The resulting pellet was resuspended, washed with PBS, and subjected to a final centrifugation step. The expression levels of miR-101b-3p in NV before electroporation and in electroporated NV (designated as NV-miR) were subsequently quantified by RT-PCR.

### 2.13 Dual-luciferase reporter system

The plasmid was designed by Taitool Bioscience. The HEK293t cells (2 × 10^3) were seeded in a 96-well plate and incubated overnight. The plasmid containing the miR-101b-3p plasmid and 3′ untranslated region (UTR) mutation (Mut) of Nav1.6 or wild-type (WT) Nav1.6 (details in Fig. 6D) was mixed with Lipofectamine™ 3000 transfection reagent (#P5667270, Invitrogen™, USA) and added to the plate for 48 h. After incubation, the cells were washed with PBS and subsequently lysed in cell lysis buffer according to the protocol of the Dual-Luciferase Reporter Assay System (DL101-01, Vazyme, China). Following cell lysis, 20 μl of the cell lysate was added to a microplate. To measure firefly luciferase activity, 100 μl of luciferase substrate was quickly dispensed into each well, and the luminescence was immediately recorded *via* a microplate reader (SyNERGY, BioTek, USA). Next, 100 μl of freshly prepared Renilla luciferase substrate working solution was added to each well, and Renilla luciferase activity was measured in the same manner.

### 2.14 Whole-cell patch-clamp recordings

DRG neurons were seeded onto a 9-mm coverslip, which was then placed in a recording chamber containing the following extracellular solution (in mM): 140 NaCl, 3 KCl, 2 CaCl_2_, 1 MgCl_2_, 10 glucose, and 10 HEPES, adjusted to PH 7.4. Electrophysiological recordings were performed *via* an Axopatch 700B amplifier and a Digidata 1440 digitizer, interfaced with a computer running pClamp 10.2 software (Molecular Devices, Sunnyvale, CA, USA). Data acquisition was carried out at a sampling rate of 20 kHz, and the data were filtered at 10 kHz. Whole-cell patch-clamp recordings were conducted on DRG neurons, and the resistances of patch electrodes ranged from 3 to 6 MΩ. The action potential (AP) was recorded in current-clamp mode with an internal solution (in mM): 126 KCH3SO3, 10 NaCl, 1 MgCl_2_, 10 HEPES, 10 EGTA, 3 Mg-ATP, and 0.3 GTP-Tris, adjusted to PH 7.3. Rheobase current was determined by applying a gradient depolarizing stimulus current (5 pA steps for 100 ms). The firing frequency was measured *via* sustained depolarizing current steps, each increasing by 5 pA for 1000 ms.

For sodium current recordings, ND7/23 cells were transfected with 50 nM miR-101b-3p mimic (Sangon Biotech, China) with a mixture of Lipofectamine™ 3000 transfection reagent (P5667270, Invitrogen™, USA) for 48 h. Following transfection, the cells were used to record *I*_Nav_ under voltage-clamping conditions. The external solution contained (in mM) 140 NaCl, 2 CaCl_2_, 2 MgCl_2_·6H_2_O, 20 TEA-Cl, 10 HEPES, and 10 glucose·H_2_O, adjusted to PH 7.4, whereas the internal solution included (in mM) 100 CsCl, 30 CsF, 10 EGTA, 10 TEA-Cl, 1 CaCl_2_, 8 NaCl, 1 MgCl_2_·6H_2_O, and 10 HEPES, adjusted to PH 7.3. The electrophysiological data were analyzed by Clampfit software 10.6 (Axon Instruments, USA).

### 2.15 Real time-qPCR

The DRG tissues were collected freshly and frozen immediately in liquid nitrogen. Before RNA extraction, the tissue was added to a lysis buffer and homogenized through magnetic bead milling within a specialized tissue homogenizer (SCIENTZ, China). The homogenized tissue was subsequently added to the centrifuge column according to the protocol of the Eastep® Super Total RNA Extraction Kit (LS1040, Promega, USA). Then, RNA was converted into cDNA through reverse transcription using HiScript IV RT SuperMix (R423-01, Vazyme, China). RT-qPCR was performed with primers and SYBR master mix (Q712-02, Vazyme, China). The primer or probe sequences are shown in Supplementary Table 1. The Ct value of the sodium channel in each DRG sample was normalized to that of β-actin. The Ct value of microRNA (miRNA) in each DRG or EV sample was normalized to U6. The relative expression of each sample was subsequently further normalized to the maximum value. The value of 2^−ΔΔCt^ was used for comparisons among groups. The sequences of all primers and probes used in this study are provided in Supplementary Table 1.

### 2.16 RNA sequencing (RNAseq) and analysis

Three EV samples were independently extracted from the supernatants of MSCs derived from three separate commercially obtained batches. C-EV and T-EV were isolated, and RNA was extracted from the EV. First, 3′ and 5’ were added to the RNA, which was then reverse-transcribed to cDNA and further amplified *via* qPCR. The resulting DNA fragments were subjected to gel extraction and purified to construct a cDNA library. The purified library was evaluated with a Qubit® 2.0 (Life Technologies, USA) or an Agilent 2100 (Agilent Technologies, USA) instrument. MiRNA sequencing was performed *via* an Illumina SE50 system (NEB, USA). Sequencing technology provides the ability to sequence and generate quantitative expression profiles for all small RNA families present in a sample. An in-depth analysis of noncoding RNAs was performed. After the raw sequencing data were subjected to quality control, the following steps were conducted: analyzing the length distribution of the clean reads; annotating small RNAs (including miRNAs, piRNAs, novel miRNAs, and ncRNA) *via* a reference genome; analyzing miRNA families; and conducting differential gene expression analysis. The edgeR algorithm was used to calculate differential miRNA expression between the two groups according to the criteria of | log2 (fold change) | ≥ 1.2 and a P value < 0.05.

### 2.17 Statistical analysis

The normality of the data distribution was evaluated *via* the Shapiro-Wilk test. The data are presented as the mean ± SEM or median (interquartile range, IQR). To compare differences between groups, the unpaired *t*-test or Mann-Whitney *U* test was applied, depending on the data distribution. Comparisons of more than two groups were performed *via* one-way analysis of variance (ANOVA) with the Bonferroni *post hoc* test or the Kruskal-Wallis with Dunn's *post hoc* test. The persistent data at different time points were analyzed by two-way repeated-measures (RM) ANOVA with the Holm-Sidak *post hoc* test. The data were analyzed with GraphPad Prism 8 (GraphPad Software, Inc.). GraphPad Prism 8 or R software (version 4.3.2) was used to draw the images. The main experimental protocols, groupings, and timelines are shown in Supplement Fig. 19, with a total of 466 mice used. A summary diagram was drawn in FigDraw and Biorender. A P-value <0.05 was considered significant.

## 3 Results

### 3.1 Cellular uptake and biodistribution of EV *in vitro* and *in vivo*

EV was extracted *via* gradient differential centrifugation and labeled with fluorescence (Fig. 1A). 50 ng/ml TNF-α was selected to precondition MSC (Supplement Fig. 1A). The characteristics of the EV, including their morphology, markers, and particle size, were validated. The markers of EV were identified *via* Western blotting, including ALIX, CD63, and CD81 (Supplementary Fig. 2B). The mean particle sizes of C‐EV and T‐EV had no difference (Supplementary Fig. 2C; n = 5 EV samples per group, 172.4 ± 7.7 nm *vs.* 165.0 ± 14.9 nm, P = 0.6721 by unpaired t‐test), and their particle size distributions were also similar (Supplementary Fig. 2E–F). The extracted EV from the supernatant of cells had a cup-shaped morphology, as identified *via* TEM (Fig. 1B; scale bar = 200 nm). Moreover, TNF-α led to a significant difference in the protein profile between T-EV and C-EV (Supplement Fig. 2 G-I).

**Fig. 1:**
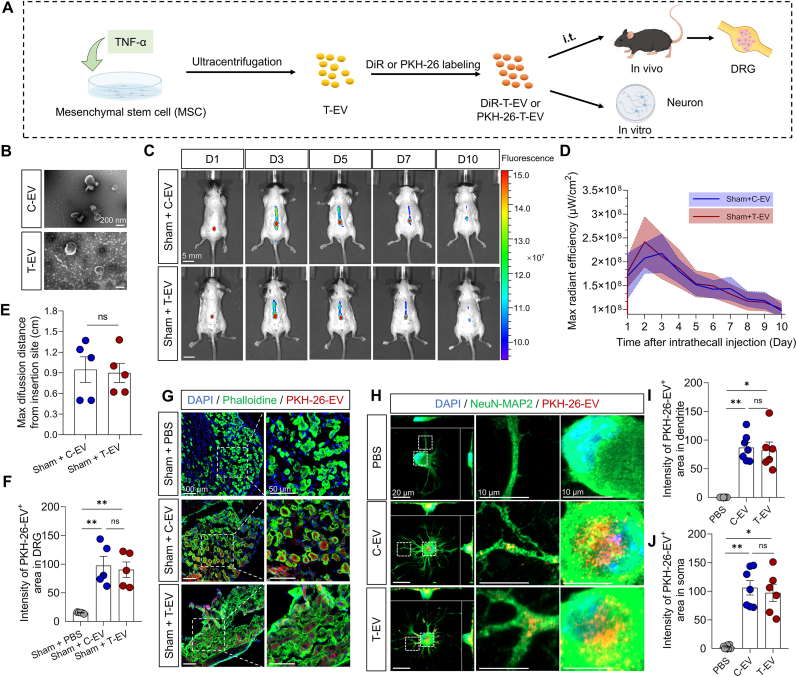
Uptake of C-EV and T-EV *in vivo* and *in vitro*. (**A**) Schematic diagram of the experiment process. (**B**) Morphology of C-EV and T-EV by TEM (scale bar, 200 nm). (C) DiR-labeled C-EV and T-EV in a mouse by *in vivo* tracing. (D) The maximum radiant efficiency of intrathecal DiR-EV (scale bar = 5 mm). (E) Maximum diffusion distance of DiR-labeled EV from the injection site on Day 2 (n = 5 mice). (**F**) Semiquantitative analysis of the PKH-26-EV^+^ area in DRG by immunofluorescence (n = 5 slices from 3 mice). (**G**) Fluorescence showing the distribution of PKH26-labeled C-EV and T-EV within DRG (scale bar, left, 100 μm, right, 50 μm). (**H**) Fluorescence images showing the internalization of PKH26-labeled C-EV and T-EV by the dendrites and somas of DRG neurons (scale bar, left, 20 μm, right, 10 μm). (**I**) Semiquantitative analysis of the EV extraction in neuronal dendrites by immunofluorescence (n = 6–7 cells). (**J**) Semiquantitative analysis of the EV extraction in neuronal soma by immunofluorescence (n = 6–7 cells). C-EV: Control mesenchymal stem cell-derived extracellular vesicle; T-EV: TNF-α-preconditioning mesenchymal stem cell-derived extracellular vesicle; TEM: Transmission electron microscope; DRG: Dorsal root ganglion; IVIS: In Vivo Imaging System. All data are presented as means ± SEM. ∗P < 0.05, ∗∗P < 0.01, ns: no significant.

To track the *in vivo* distribution, EV was labeled with DiR and then intrathecally delivered to a mouse. DiR-labeled C‐EV and T‐EV were observed to distribute and diffuse along the spinal cord (Fig. 1C) and were gradually metabolized over 10 days following injection. There were no significant differences between the groups in maximum radiant efficiency (Fig. 1D; n = 5 mice per group, F (1, 8) = 0.009, P = 0.9273 by two-way ANOVA) or diffusion distance from injection site (Fig. 1E; 0.95 ± 0.19 cm *vs*. 0.90 ± 0.14 cm, P = 0.8424 by unpaired t‐test). C-EV and T-EV were also labeled with PKH-26, a red fluorescent membrane dye, to enable direct visualization of their interaction with neurons. Next, we investigated the uptake of PKH-26-labeled EV by DRG cells *in vivo*. The results demonstrated that both C-EV and T-EV were successfully internalized by DRG cells (Fig. 1F–G; n = 5 slices from 3 mice per group, PBS *vs.* C-EV, P = 0.0013; PBS *vs.* T-EV, P = 0.0026 by one-way ANOVA followed by Bonferroni *post hoc* test), with no significant differences observed in fluorescence area between the C‐EV and T‐EV groups. Both PKH-26-labeled EVs were also internalized by the spinal dorsal horn (SDH; Supplementary Fig. 3A–C), but not by the sympathetic preganglionic neurons of the intermediolateral nucleus (IML; Supplementary Fig. 3D–F). To further investigate the uptake of EV in neurons, primary DRG neurons were isolated and incubated with PKH-26-labeled C-EV and PKH-26-labeled T-EV. Fluorescence images revealed clear internalization of C-EV and T-EV in primary neurons (Fig. 1H). The PKH-26-labeled EVs were internalized by the dendrites (Fig. 1H–I; n = 6–7 cells per group; PBS *vs.* C-EV, P < 0.001; PBS *vs.* T-EV, P < 0.001 by one-way ANOVA followed by Bonferroni *post hoc* test) and soma (Fig. 1H and J; PBS vs. C-EV, P = 0.0028; PBS vs. T-EV, P = 0.0113 by Kruskal-Wallis test followed by Dunn's *post hoc* test), but there was no difference in the fluorescence area between C-EV and T-EV groups. These results suggest that DRG neurons are capable of internalizing equal amounts of C-EV and T-EV.

### 3.2 T-EV has better analgesic effects than C-EV

The application of EV at concentrations of 0, 5, and 10 μg resulted in a dose-dependent increase in the mechanical threshold. However, no significant difference was observed when T-EV was applied at concentrations of 10 μg and 20 μg (Supplementary Fig. 4; n = 5–6 mice per group, Kruskal-Wallis followed by Dunn's *post hoc* test). Based on these findings, a concentration of 10 μg was selected for subsequent experiments. Furthermore, neither C-EV nor T-EV affected mechanical sensitivity or thermo-sensation in *naïve* mice (Supplementary Fig. 5A–E; n = 5 mice per group; one-way ANOVA followed by Bonferroni *post hoc* test or Kruskal-Wallis followed by Dunn's *post hoc* test). The motor function (movement distance and velocity in Supplementary Fig. 5F–G; n = 6 mice per group, paired *t*-test) was not influenced by EV injection. Intrathecal EV administration did not affect the morphology of the DRG or spinal cord, and no necrosis was observed in either DRG or superficial spinal cord regions (Supplement Fig. 5H–I).

The treatment was administered during the early phase of the CCI model, from day 3 to day 5 post-surgery. For the behavioral test, three stimulants were employed: brush, von-Frey, and pinprick, each representing different intensities of mechanical sensory input. In the brush test, light mechanical stimulation was applied. The von-Frey test was used to assess sensitivity to punctate mechanical stimulation, and the pinprick test was used to evaluate the response to noxious stimulation. Compared with C-EV treatment, T-EV treatment reduced the brush response compared to C-EV (Fig. 2C; n = 11 mice per group, 60 % [IQR 30 %] for C-EV *vs.* 30 % [IQR 20 %] for T-EV, P = 0.043 by Kruskal-Wallis followed by Dunn's *post hoc* test). Additionally, T-EV reversed the mechanical response from Day 4 to Day 18 (Fig. 2B; P < 0.0001 by two-way RM ANOVA followed by Holm-Sidak *post hoc* test), with a complete reversal of the mechanical threshold observed on Day 6; however, C-EV had no such effect (C-EV *vs.* T-EV, n = 11 per group, P < 0.001 on Day 6). The normalized area under the curve (AUC) of the persistent von-Frey test in the T-EV group was greater than that in the C-EV group (Fig. 2B right panel; 33 % ± 0.82 % *vs.* 14.86 % ± 0.49 %, P < 0.0001 by unpaired *t*-test). The withdrawal latency in response to the pinprick test was also significantly reduced following T-EV delivery compared to C-EV delivery (Fig. 2D; n = 11 per group, 723.6 ± 49.11 ms *vs.* 1239 ± 81.18 ms, P < 0.001 by one-way ANOVA followed by Bonferroni *post hoc* test). Compared with C-EV treatment, T-EV treatment also decreased noxious heat and cold sensitivity in the models (Fig. 2E–F; n = 11 mice per group, two-way RM ANOVA followed by Holm-Sidak *post hoc* test). Moreover, both C-EV and T-EV improved the movement function of the right hindlimb (Fig. 2I–L, n = 11–12 mice per group). However, there were no significant differences between the C-EV and T-EV groups in the ipsilesional-to-contralesional hindlimb standing ratio (73.78 % [IQR 45.97 %] *vs.* 81.76 % [41.41 %], P > 0.05), mean print area (95.83 % [IQR 52.39 %] *vs*. 85.93 % [IQR 73.87 %], P > 0.05), or mean print intensity (92.47 % [IQR 10.16 %] *vs.* 95.85 % [IQR 17.43 %], P > 0.05 by Kruskal-Wallis followed by Dunn's *post hoc* test). The latency (9.41 ± 0.75 ms *vs.* 8.42 ± 0.43 ms, P > 0.05 by one-way ANOVA followed by Bonferroni's post hoc test) of somatosensory evoked potential (SEP) and sciatic nerve conduction velocity (8.05 ± 0.60 m/s *vs.* 8.58 ± 0.40 m/s, P > 0.05 by one-way ANOVA followed by Bonferroni's post hoc test) between C-EV and T-EV groups also had no difference (Supplement Fig. 7C-D, n = 10 mice per group). These findings suggest that T-EV treatment further alleviates both mechanical and thermal sensitivity-induced pain, but does not further reverse the motor function and nerve conduction velocity.

**Fig. 2:**
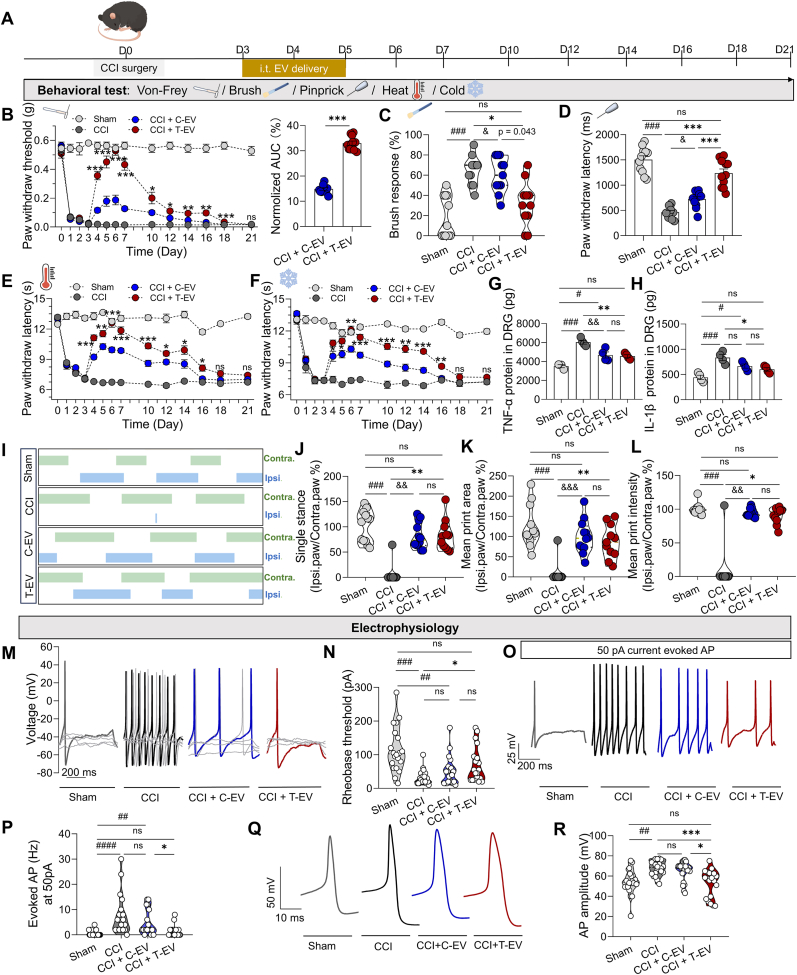
Effect of EV treatment on pain behavior. (**A**) Schematic timeline of the experimental design. (**B-D**) Mechanical hypersensitivity assessment following intrathecal administration of C-EV or T-EV (n = 11 mice): (B) von-Frey test (left panel); normalized AUC of C-EV and T-EV groups (right panel); (C) brush test, (D) pinprick test. (**E**) Paw withdrawal latency of the Hargreaves test (n = 11 mice). (**F**) Paw withdrawal latency of dry ice-induced cold stimulation (n = 11 mice per group). (**G-H**) Expression of TNF-α and IL-1β in DRG on Day 6 by ELISA (n = 3–4 mice). (**I**) Representative example of mouse hindlimb gait. (**J**) Ratio of single-limb stance duration (ipsilesional/contralesional). (**K**) Ratio of hindlimb contact area (ipsilesional/contralesional) (**L**) Ratio of mean print intensity (ipsilesional/contralesional) (n = 10 mice). (**M**) Sample of rheobase threshold. (**N**) Summary data of rheobase thresholds (n = 20–22 cells from 3 mice). (**O**) Sample of AP in response to 50 pA current stimulation. (**P**) Summary data of AP firing frequency (n = 17–19 cells from 3 mice). (**Q**) Sample of AP amplitude. (**R**) Summary data of AP amplitudes (n = 20–22 cells from 3 mice). C-EV: Control mesenchymal stem cell-derived extracellular vesicle; T-EV: TNF-α-preconditioning mesenchymal stem cell-derived extracellular vesicle; AUC: Area under the curve; DRG: Dorsal root ganglion; AP: Action potential; ELISA: Enzyme-linked immunosorbent assay. All data are expressed as means ± SEM or median (IQR). ∗P < 0.05, ∗∗P < 0.01, ∗∗∗P < 0.001, ^#^P < 0.05, ^##^P < 0.01, ^###^P < 0.001, ^&^P < 0.05, ^&&^P < 0.01, ^&&&^P < 0.001, ns: no significant.

To elucidate the detailed mechanism underlying the enhanced pain relief of T-EV, we assessed the expression of inflammatory mediators in the DRG following EV administration. ELISA analysis revealed that T-EV treatment did not significantly alter the levels of TNF-α (T-EV *vs.* C-EV: 4560.00 ± 155.10 ng *vs.* 4688.00 ± 322.60 ng, P > 0.05), IL-1β (T-EV *vs.* C-EV: 601.80 ± 35.77 ng *vs.* 666.70 ± 41.52 ng, P > 0.05), IL-6 (T-EV *vs.* C-EV: 715.40 ± 48.95 ng *vs.* 815.30 ± 25.52 ng, P = 0.067), IL-10 (T-EV *vs.* C-EV: 2316.00 ± 129.40 ng *vs.* 1848.00 ± 33.55 ng, P = 0.111), or CGRP (T-EV *vs.* C-EV: 254.90 ± 11.96 ng *vs.* 250.60 ± 5.93 ng, P > 0.05) in DRG tissues compared with the C-EV group (Fig. 2G–H and Supplementary Fig. 6A–C; n = 3–4 mice per group, one-way ANOVA followed by Bonferroni's post hoc test). These results indicated that the enhanced analgesic effect of T-EV was not primarily attributable to the modulation of neuroinflammation. We subsequently assessed the direct effect of EV on the function of DRG neurons. After EV treatment *in vivo*, we observed changes in the rheobase current of the different groups *via* electrophysiological recordings (Fig. 2I–J). T-EV increased the rheobase threshold on day 6 after CCI surgery (n = 20–22 cells from 3 mice per group, 25.00 [IQR 22.50] pA *vs.* 50.00 [IQR 67.50] pA, P = 0.0421 by Kruskal-Wallis followed by Dunn's *post hoc* test), but there was no difference between T-EV and C-EV treatment (50.00 [IQR 67.50] pA *vs.* 45.00 [IQR 42.50] pA, P > 0.05). However, compared with C-EV, T-EV decreased the AP firing frequency under 50 pA current stimulation (Fig. 2K and L; n = 17–19 cells from 3 mice per group, P = 0.0475 by Kruskal-Wallis followed by Dunn's *post hoc* test). Moreover, compared with that in the C-EV treatment group, the AP amplitude in the T-EV-treated group was also reversed (Fig. 2M and N; n = 20–22 cells from 3 mice per group, 67.64 [IQR 9.82] mV *vs.* 57.89 [IQR 25.08] mV, P = 0.0140 by Kruskal-Wallis followed by Dunn's *post hoc* test). These results suggested that T-EV had a better analgesic effect and inhibited the excessive excitability of DRG neurons.

### 3.3 Differential miRNA expressions in T-EV and C-EV

MicroRNAs (miRNAs) have emerged as critical molecular regulators in the biological processes of MSC-EV [41,42]. To investigate the role of miRNAs in EV-mediated effects, we performed miRNA sequencing to identify differential miRNA expression between C-EV and T-EV. This analysis aimed to elucidate the potential mechanisms underlying the enhanced analgesic effects of T-EV. A total of 39 miRNAs were found to be upregulated in T-EV compared to C-EV (Supplementary Fig. 8). The proportional distribution of differentially expressed miRNAs was presented in Fig. 3A, with let-7i-5p, miR-143–3p, miR-101b-3p, miR-148–3p, and miR-451a representing the most abundant miRNAs (collectively 80 % of total miRNA content). The top 10 differentially expressed miRNAs were displayed in a heatmap (Fig. 3B). RT-qPCR was used to validate the elevated expression of miRNA among these high-abundance miRNAs in T-EV, and confirmed that miR-101b-3p, miR-143–3p, and let-7i-5p were elevated in T-EV (Fig. 3C; n = 3–4 per group, unpaired *t*-test). Furthermore, among these top 10 miRNAs, three (let-7i-5p, miR-101b-3p, and miR-451a) exhibited markedly greater differential expression between groups according to log2 (fold change) > 1.2 (Fig. 3D). Notably, previous reports have indicated that the miR-101b-3p level was decreased in patients with neuropathic pain [26]. Therefore, let-7i-5p, miR-101b-3p, and miR-451a were used to validate in the next experiment.

**Fig. 3:**
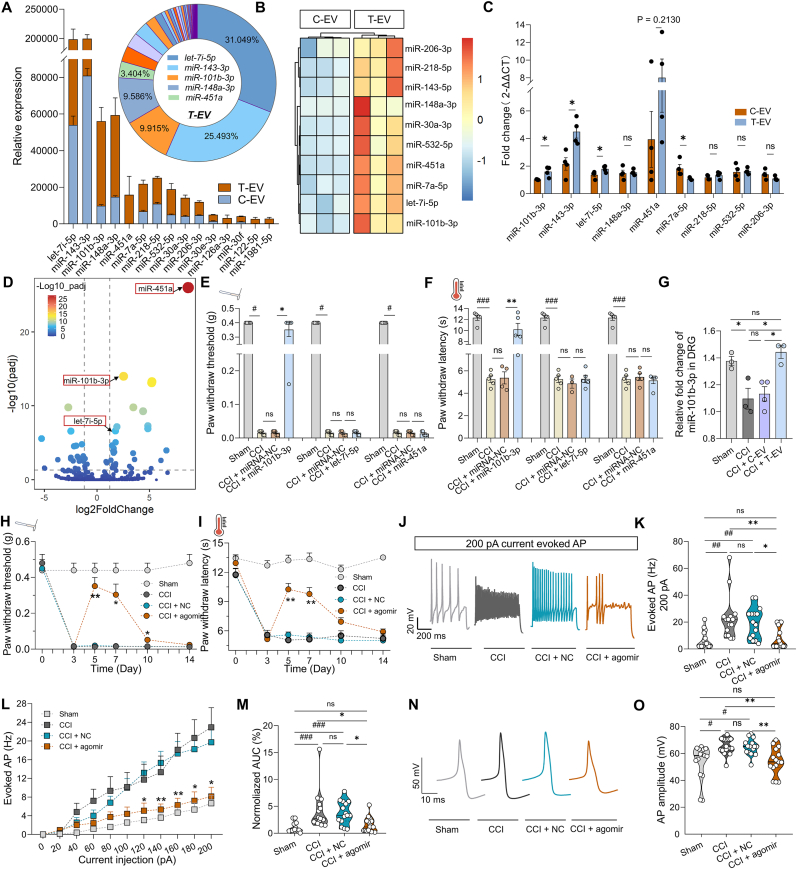
Analysis of differential miRNAs between C-EV **and T-EV.** (**A**) Relative expression of the top 15 differential miRNAs in C-EV and T-EV, and the proportion of highly expressed miRNAs among the differential genes in T-EV. (**B**) Heatmap of the top 10 differentially expressed miRNAs based on sequence analysis in C-EV and T-EV. (**C**) Validation of differential miRNA expression in C-EV and T-EV by RT-qPCR (n = 3–4 EV samples). (**D**) Volcano plot depicting differential miRNA expressions. Right: increased miRNA in T-EV. Left: decreased miRNA in T-EV. (**E**) Paw withdrawal threshold following miR-101b-3p, let-7i-5p, and miR-451a intrathecal delivery separately (n = 4–5 mice per group). (**F**) Paw withdrawal latency of the Hargreaves test following miR-101b-3p, let-7i-5p, and miR-451a intrathecal delivery separately. (**G**) Relative expression of miR-101b-3p in DRG by RT-qPCR (n = 3–4 mice). (**H-I**) Von-Frey and Hargreaves test after miR-101b-3p agomir intrathecally delivery (n = 4–5 mice). (**J**) Sample of AP in response to 200 pA current injection. (**K**) Summary data of AP firing frequency. (**L**) Summary data of AP firing numbers under 0–200 pA gradient current stimulation (n = 15 cells from 3 mice per group). (**M**) AUC (normalized to the sham group) in different groups. (**N**) Sample of AP amplitude. (**O**) Summary data of AP amplitudes (n = 15 cells from 3 mice per group). DRG: Dorsal root ganglion; C-EV: Control mesenchymal stem cell-derived extracellular vesicle; T-EV: TNF-α-preconditioning mesenchymal stem cell-derived extracellular vesicle; AUC: Area under the curve; AP: Action potential. All data are expressed as means ± SEM or median (IQR). ∗P < 0.05, ∗∗P < 0.01, ^#^P < 0.05, ^##^P < 0.01, ^###^P < 0.001, ns: no significant.

### 3.4 MiR-101b-3p has an analgesic effect on neuropathic pain

To determine whether the three above miRNAs had analgesic effects, the pain behavior tests were used to verify the effects of intrathecally delivering their agomir separately. Notably, miR-101b-3p demonstrated significant analgesic effects in the CCI model (Fig. 3E and F; n = 5 mice per group, CCI + miRNA-NC *vs.* CCI + miR-101b-3p, 0.02 [IQR 0.012] g *vs.* 0.4 [IQR 0.12] g, P = 0.04 by Kruskal-Wallis followed by Dunn's *post hoc* test for von-Frey test; 5.38 ± 0.54 ms *vs.* 10.20 ± 1.11 ms, P = 0.0013 by one-way ANOVA followed by Bonferroni *post hoc* test for Hargreaves test). But the other let-7i-5p and miR-451a had no analgesic effect (Fig. 3E and F; n = 4–5 mice per group, Kruskal-Wallis followed by Dunn's *post hoc* test for von-Frey test and one-way ANOVA followed by Bonferroni *post hoc* test for Hargreaves test). Moreover, RT-qPCR analysis revealed that miR-101b-3p expression in DRG was decreased in the CCI group (Fig. 3G; n = 3–4 mice per group, P = 0.0466 by one-way ANOVA followed by Bonferroni post hoc test), and compared with C-EV, T-EV reversed this decrease (Fig. 3G; P = 0.0174), suggesting that T-EV restored miR-101b-3p expression in the DRG, but C-EV did not significantly reverse it. These findings collectively demonstrate that miR-101b-3p may contribute to the enhanced analgesic effects of T-EV. To determine whether TNF-α induces similar changes in human BMSC, hBMSC was preconditioned with equal amounts of TNF-α and found that miR-101b-3p was also significantly upregulated in T-EV derived from these human cells (Supplement Fig. 9B; n = 5 EV samples per group, P = 0.015 by one-way ANOVA followed by Bonferroni *post hoc* test). TNF-α is known to activate the downstream NF-κB signaling pathway. To determine whether TNF-α enhances miR-101b-3p expression in T-EV through this pathway, we suppressed NF-κB signaling in TNF-α-preconditioning MSC. The fold change of miR-101b-3p in the NF-κB inhibitor (IN-11) group was 1.63 ± 0.24 (Supplement Fig. 10B), which was decreased compared to the T-EV group (2.81 ± 0.31, P = 0.025 by one-way ANOVA followed by Bonferroni *post hoc* test, n = 3–4 EV samples per group).

Furthermore, to investigate the effects of miR-101b-3p on the AP firing rate and amplitude, electrophysiological recordings were performed to assess changes in DRG neurons following the administration of the miR-101b-3p agomir. Compared with that in the NC group, the mechanical analgesia induced by the miR-101b-3p agomir persisted until day 10 (Fig. 3H; n = 4–5 mice per group, two-way RM ANOVA followed by Holm-Sidak *post hoc* test). However, the effect on heat pain sensitivity was only sustained up to Day 7 (Fig. 3I; n = 4–5 mice, two-way RM ANOVA followed by Holm-Sidak *post hoc* test). Whole-cell patch-clamp recordings further revealed that agomir treatment significantly inhibited action potential (AP) firing in response to a maximal 200 pA current stimulus (Fig. 3J–K; n = 15 cells from 3 mice, P = 0.0103, Kruskal-Wallis test followed by Dunn's *post hoc* test). Additionally, agomir-treated neurons presented a reduced AP firing rate in response to gradient current injections (Fig. 3L; n = 15 cells from 3 mice per group, two-way RM ANOVA followed by Holm-Sidak *post hoc* test). The normalized AUC for the AP firing rate was significantly lower in the agomir-treated group than in the NC group (Fig. 3M; 1.16 % [IQR 1.78 %] *vs.* 3.33 % [IQR 3.63 %], P = 0.0124 by Kruskal-Wallis test followed by Dunn's *post hoc* test). Furthermore, miR-101b-3p reversed the AP amplitude compared with that of the NC group after CCI surgery (Fig. 3N–O; n = 15 cells from 3 mice per group, 53.74 [IQR 13.67] mV *vs.* 65.25 [IQR 7.20] mV, P = 0.0094 by Kruskal-Wallis followed by Dunn's *post hoc* test), suggesting a potential modulatory effect of miR-101b-3p on voltage-gated sodium channels (VGSCs). Collectively, these results demonstrate that miR-101b-3p directly modulates neuronal excitability, likely through the regulation of VGSCs.

### 3.5 Knockdown of miR-101b-3p in T-EV abolishes the enhanced analgesic effect

Based on these findings, we further investigated the role of miR-101b-3p in the enhanced analgesic effects of T-EV by silencing miR-101b-3p in T-EV. MSC was transfected with VSVG-LENTAI-hU6-shRNA (miR-101b-3p)-esEF1A-MataGFP-IRES-PuroR-WPRE-pA, and puromycin selection was used to eliminate untransfected cells, ensuring efficient knockdown. The transfection efficiency was subsequently confirmed through fluorescence imaging (Fig. 4B) and flow cytometry (Fig. 4C; n = 5 dishes per group). RT-qPCR analysis revealed a significant reduction in miR-101b-3p expression in the T-EV-shRNA-miR-101b-3p (T-EV-shRNA) group (Fig. 4D; n = 5 EV samples per group, P = 0.0139 by Kruskal-Wallis test followed by Dunn's *post hoc*). The analgesic effect of T-EV-shRNA was subsequently tested *in vivo*. The results of the von-Frey test revealed that T-EV-NC increased the mechanical threshold, but after silencing miR-101b-3p in T-EV, T-EV-shRNA did not have the effect (Fig. 4F; n = 8 mice per group, two-way RM ANOVA followed by Holm-Sidak *post hoc* test). Furthermore, the normalized AUC for T-EV-shRNA (Fig. 4F; 19.38 % ± 1.21 %) was comparable to that of C-EV (23.10 % ± 1.51 %; P = 0.1008, unpaired *t*-test) (Fig. 2B), suggesting that silencing miR-101b-3p in T-EV produced an analgesic effect similar to that of C-EV. Moreover, compared with T-EV-shRNA treatment, T-EV-NC treatment increased the pinprick response time on day 6 post-CCI surgery (Fig. 4G; n = 8 mice per group, 652.50 ± 42.04 ms *vs.* 1141.00 ± 119.20 ms, P = 0.0046 by one-way ANOVA followed by Bonferroni *post hoc* test). Similarly, silencing miR-101b-3p in the T-EV-shRNA group significantly attenuated the analgesic effect in the Hargreaves test compared to the T-EV-NC group (Fig. 4H; n = 8 mice per group, two-way RM ANOVA followed by Holm-Sidak *post hoc* test). Additionally, T-EV-NC treatment reversed cold pain induced by dry ice on day 6 after surgery, in contrast to T-EV-shRNA delivery (Fig. 4I; n = 7–8 mice per group, 12.00 [IQR 2.50] s *vs.* 7.28 [IQR 0.50] s, P = 0.0409 by Kruskal-Wallis followed by Dunn's *post hoc* test). Overall, silencing miR-101b-3p reduced the enhanced analgesic effect of T-EV on noxious mechanical and thermal pain. These findings indicate that miR-101b-3p is crucial for the enhanced analgesic effects of T-EV.

**Fig. 4:**
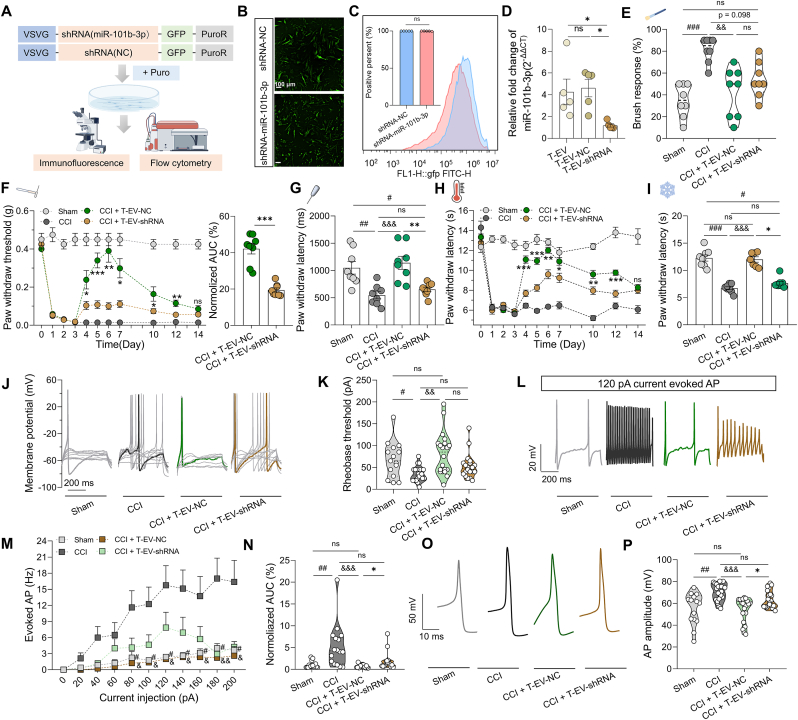
MiR-101b-3p mediates the enhanced analgesic effec**t of T-EV.** (**A**) Schematic of miR-101b-3p knockdown in MSC. (**B**) Fluorescence image of virus transfection in MSC (scale bar, 100 μm). (**C**) Efficiency of virus infection assessed by flow cytometry (n = 5 dishes). (**D**) The expression of miR-101b-3p in T-EV, T-EV-shRNA-NC, and T-EV-shRNA-miR-101b-3p groups by RT-qPCR (n = 5 EV samples). (**E-G**) Mechanical sensitivity test in mice (n = 8 mice): (E) brush test, (F) von-Frey test (left panel), normalized AUC of C-EV and T-EV groups (right panel), (G) pinprick test. (**H**) Paw withdrawal latency of the Hargreaves test (n = 8 mice). (**I**) Latency to withdrawal of dry ice-induced cold stimulation (n = 7–8 mice). (**J**) Sample of rheobase threshold. (**K**) Summary data of rheobase thresholds (n = 15–20 cells from 3 mice). (**L**) Sample of AP firing. (**M**) Summary data of AP firing numbers under 0–200 pA current injections (n = 15–16 cells from 3 mice). (**N**) Summary data of AUC (normalized to the sham group) in different groups. (**O**) Sample of AP amplitude. (**P**) Summary data of AP amplitude (n = 15–20 cells from 3 mice). C-EV: Control mesenchymal stem cell-derived extracellular vesicle; T-EV: TNF-α-preconditioned mesenchymal stem cell-derived extracellular vesicle; CCI: Chronic constriction injury model; AP: Action potential; AUC: Area under the curve. All data are expressed as means ± SEM or median (IQR). ∗P < 0.05, ∗∗P < 0.01, ∗∗∗P < 0.001, ^#^P < 0.05, ^##^P < 0.01, ^###^P < 0.001, ^&&^P < 0.01, ^&&&^P < 0.001, ns: no significant.

We next investigated the role of miR-101b-3p in mediating the effects of T-EV on DRG neurons. No significant difference was observed in the rheobase threshold between the T-EV-shRNA and T-EV-NC groups (Fig. 4J–K; n = 15–20 cells from 3 mice per group, P = 0.4176, Kruskal-Wallis test followed by Dunn's *post hoc* test). While T-EV-NC treatment decreased the AP firing rate compared with that of the CCI group, silencing miR-101b-3p in T-EV reversed this effect (Fig. 4L–M; n = 15–16 cells from 3 mice per group, two-way RM ANOVA followed by Holm-Sidak *post hoc* test). The normalized AUC of AP firing frequency was significantly lower in the T-EV-shRNA group compared to the T-EV-NC group (Fig. 4N; 1.27 % [IQR 1.23 %] *vs.*0.59 % [IQR 0.39 %], P = 0.0339 by Kruskal-Wallis test followed by Dunn's *post hoc* test), suggesting that silencing miR-101b-3p induced a decrease in the AP firing frequency in T-EV. Furthermore, T-EV-NC treatment decreased the AP amplitude, whereas no such effect was observed in the T-EV-shRNA group (Fig. 4O–P; n = 15–20 cells from 3 mice per group, 58.76 [IQR 18.48] mV *vs.* 61.47 [IQR 13.57] mV, P = 0.0203 by Kruskal-Wallis followed by Dunn's *post hoc* test). These electrophysiology recordings demonstrated that miR-101b-3p modulates the effect of T-EV on neuronal hyperexcitability.

### 3.6 Voltage-gated sodium channels as potential targets of miR-101b-3p

To identify potential targets of miR-101b-3p, we utilized several prediction databases, including TargetScan, miRDB, and miRWALK, which suggested that VGSCs might be targets of miR-101b-3p (Fig. 5A). We next assessed the expression of the genes involved in pain processing, specifically Scn8a (encoding Nav1.6), Scn9a (encoding Nav1.7), and Scn10a (encoding Nav1.8), in the DRG *via* RT-qPCR. Compared to the sham group (Fig. 5B), fold changes were 1.55 ± 0.09 on day 3 (*vs.* 1.06 ± 0.04, P = 0.0300) and 1.77 ± 0.15 on day 6 (*vs.* 1.06 ± 0.04, P = 0.0024 by one-way ANOVA followed by Bonferroni *post hoc* test). However, no significant changes were observed for Scn9a or Scn10a (Fig. 5C and D; n = 4–6 mice per group). The fluorescence image revealed that the expression of Nav1.6 was also decreased (Fig. 5E and F; n = 8 slices from 3 mice per group; sham *vs.* D3, P < 0.0001; sham *vs.* D6, P < 0.0001 by one-way ANOVA followed by Bonferroni *post hoc* test). The expression of Nav1.7 and Nav1.8 did not change (Fig. 5G–J; one-way ANOVA followed by Bonferroni *post hoc* test). Then the I_*Nav*_ current was recorded in ND7/23 cells by whole-cell patch-clamping recording (Fig. 5K). ND7/23 cells mainly expressed Nav1.6 and Nav1.7 [43]. The currents density was decreased after miR-101b-3p mimic transfection compared with those in the NC group (Fig. 5L and M; n = 14–15 cells per group, −67.07 [IQR 79.31] pA/pF *vs.* −106.9 [IQR 94.73] pA/pF, P = 0.0175 by Kruskal-Wallis followed by Dunn's *post hoc* test). These results suggest that Nav1.6 is the potential target of miR-101b-3p in the CCI model.

**Fig. 5:**
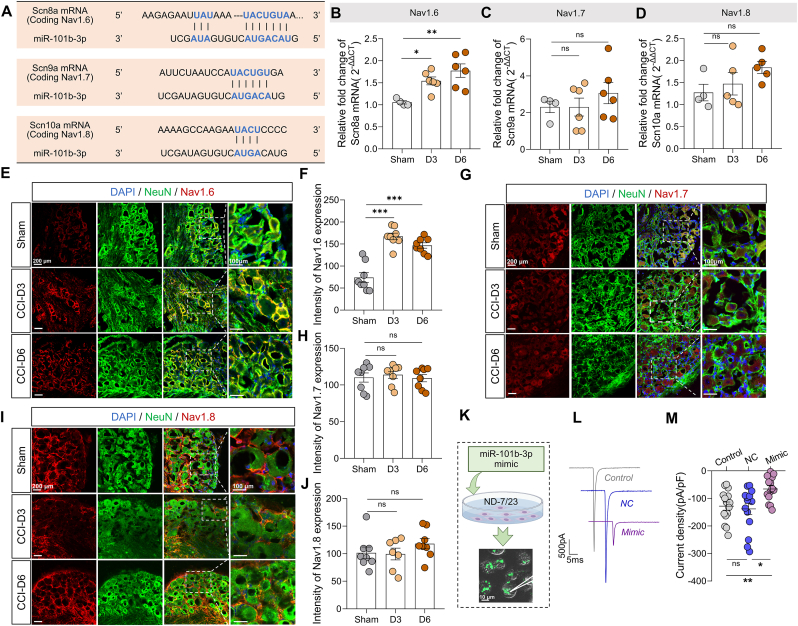
Potential targets of mi**R-101b-3p.** (**A**) Predicted miR-101b-3p target sites in Nav1.6, Nav1.7, and Nav1.8. (**B**) Relative Scn8a mRNA expression (encoding Nav1.6) after CCI surgery on Day 3 and Day 6 by RT-qPCR (n = 4–6 mice). (**C**) Relative Scn9a mRNA expression (encoding Nav1.7) after CCI surgery on Day 3 and Day 6 by RT-qPCR (n = 4–6 mice). (**D**) Relative Scn10a mRNA expression (encoding Nav1.8) after CCI surgery on Day 3 and Day 6 by RT-qPCR (n = 4–5 mice). (**E-F**) Semiquantitative analysis of Nav1.6 expression in DRG by immunofluorescence (n = 8 slices from 3 mice, scale bar, left, 200 μm, right, 100 μm). Red color indicates Nav1.6 expression, and green color indicates NeuN expression. (**G-H**) Semiquantitative analysis of Nav1.7 expression in DRG by immunofluorescence (n = 8 slices from 3 mice). (**I-J**) Semiquantitative analysis of Nav1.8 expression in DRG by immunofluorescence (n = 7–8 slices from 3 mice). (**K**) Diagram showing the electrophysiological recordings after miR-101b-3p transfection into ND-7/23 cells (scale bar, 10 μm). (**L**) Representative trace of I_*Nav*_. (**M**) Summary data of I_*Nav*_ current density (n = 14–15 cells). DRG: dorsal root ganglion; CCI: Chronic constriction injury model. All data are expressed as means ± SEM. ∗P < 0.05, ∗∗P < 0.01, ∗∗∗P < 0.001 ns: no significant.

### 3.7 Identification of Nav1.6 as a direct target of miR-101b-3p

To further confirm Nav1.6 as the main target of miR-101b-3p, we administered miR-101b-3p agomir in the CCI model and analyzed its effects on sodium channel expression in the DRG. The protein sequencing of DRG between the CCI and agomir treatment models found the decreased expression of Nav1.6 in the agomir treatment group (Supplement Fig. 11A–B). Agomir treatment also significantly reduced the expression of Nav1.6 on Day 6 after CCI surgery by immunofluorescence (Fig. 6A and B; n = 8–9 slices from 3 mice per group, P = 0.0001 by one-way ANOVA followed by Bonferroni *post hoc* test), whereas no significant changes in Nav1.7 or Nav1.8 expression were detected (Supplementary Fig. 12A–D). Moreover, compared with C-EV treatment, T-EV treatment significantly decreased Nav1.6 expression in DRG neurons (Supplementary Fig. 13; n = 12 cells per group, P = 0.0316 by Kruskal-Wallis followed by Dunn's *post hoc* test). The target site of Scn8a, encoding Nav1.6, was found to be conserved across species (Fig. 6C). There are three potential target sites in the Nav1.6 3′UTR for miR-101b-3p (Fig. 6D). To confirm direct interaction, we constructed a plasmid containing mutations at all three miR-101b-3p target sites within the Nav1.6 3′UTR. Compared with that in the NC group, the relative luciferase activity in the WT Nav1.6 group was significantly lower (Fig. 6G; n = 8 dishes per group, P = 0.0002 by unpaired *t*-test), whereas no significant change was detected in the Mutant Nav1.6 group (Fig. 6J; n = 8 dishes per group, P = 0.2203 by unpaired *t*-test). These results indicated that Nav1.6 was the direct target of miR-101b-3p. Nav1.6 expression was detected in IB4^+^, NF200^+^, and CFRP^+^ neurons, with expression levels of 4.59 % ± 1.14 %, 17.80 % ± 1.37 %, and 15.46 % ± 4.11 % respectively (Supplementary Fig. 14A–B; n = 4–5 slices from 4 to 5 mice per group). To further explore the functional role of Nav1.6 in DRG neurons, we delivered a siRNA targeting Nav1.6 to the L4 and L5 DRGs. This treatment significantly reduced Nav1.6 mRNA expression in the CCI model (Supplementary Fig. 14E; n = 3–4 mice per group, P = 0.0289 by unpaired *t*-test). Importantly, the downregulation of Nav1.6 led to an increase in mechanical threshold in the CCI model (Supplementary Fig. 14F; n = 5 mice per group, P = 0.0079 by Mann-Whitney test), indicating that reduced Nav1.6 expression may diminish pain sensitivity.

**Fig. 6:**
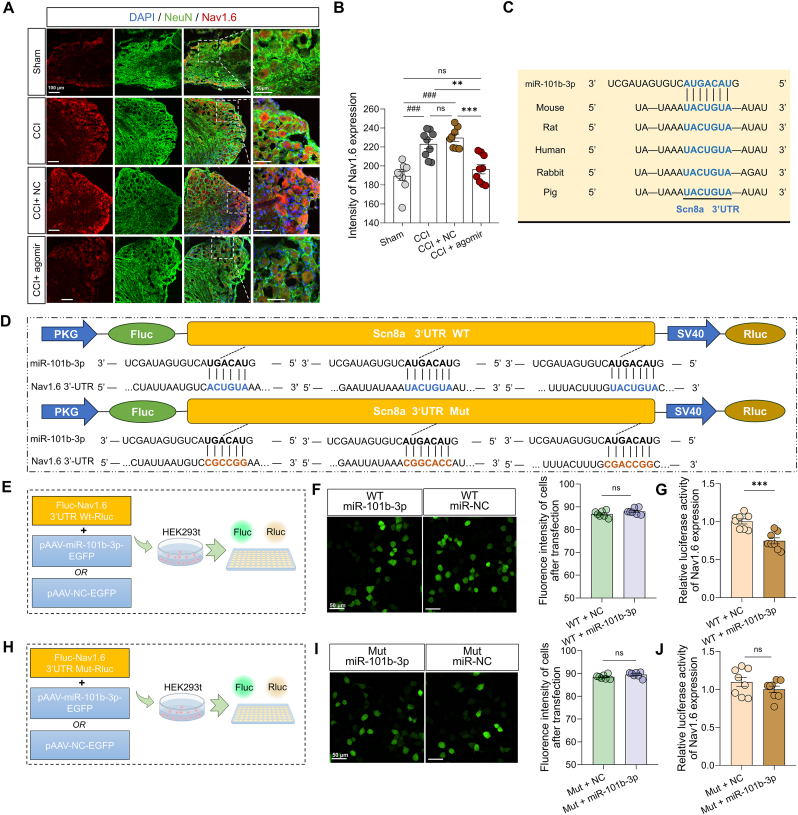
MiR-101b-3p targets to regulate Nav1.6 e**xpression.** (**A**) Fluorescence image of Nav1.6 expression in DRG after intrathecal delivery of miR-101b-3p agomir (scale bar, left, 100 μm, right, 50 μm). Red color indicates Nav1.6 expression, and green color indicates NeuN expression. (**B**) Semiquantitative analysis of Nav1.6 expression in DRG by immunofluorescence (n = 8–9 slices from 3 mice). (**C**) Conserved miR-101b-3p binding sites in the 3′UTR of the Scn8a gene across different species. (**D**) Schematic representation of identification and mutation of three miR-101b-3p target sites in the 3′ UTR of Nav1.6. (**E**) Schematic diagram of the experimental process. (**F**) Fluorescence images and semiquantitative analysis of HEK-293t cells transfected with wt Nav1.6 and miR-101b-3p plasmid (scale bar: 20 μm). (**G**) Effect of miR-101b-3p plasmid on relative luciferase activity in HEK-293t cells transfected with wt Nav1.6 (n = 8 dishes). (**H**) Schematic diagram of the experimental process. (**I**) Fluorescence images and semiquantitative analysis of HEK-293t cells transfected with mut-Nav1.6 and miR-101b-3p plasmid (scale bar: 50 μm). (**J**) Effect of miR-101b-3p plasmid on relative luciferase activity in HEK-293t cells transfected with mutant Nav1.6 (n = 8 dishes). CCI: Chronic constriction injury model; DRG: Dorsal root ganglion; HEK-293t: Human embryonic kidney 293t cell; 3′UTR: 3′Untranslated region; WT: Wild type; Mut: Mutation. All data are expressed as means ± SEM. ^###^P < 0.001, ∗∗P < 0.01, ∗∗∗P < 0.001, ns: no significant.

### 3.8 NV-encapsulated miR-101b-3p produces potent analgesic effects

To increase the yield of EV loaded with miR-101b-3p, we isolated NV from MSC *via* extrusion through an Avanti® MiniExtruder. This method is convenient, efficient, and yields high output. Next, we upregulated the expression of miR-101b-3p in NV derived from MSC. MiR-101b-3p was loaded into the NV *via* electroporation, and RT-qPCR analysis demonstrated that transfection significantly increased the fold change of miR-101b-3p in NV (4.12 ± 0.48) compared with the pre-transfection level (1.14 ± 0.22) (Fig. 7C; n = 3 NV samples per group, P = 0.024 by paired *t*-test). To quantify the concentration of miR-101b-3p in NV following transfection, miR-101b-3p was labeled with a Cy5 fluorescent marker. Consistently, the concentration of Cy5-miR-101b-3p was significantly higher after transfection than before (Supplementary Fig. 15B; n = 5 NV samples, P = 0.0011 by paired *t*-test). These results demonstrated the efficacy of electroporation. The mean particle size of NV and NV-miR was similar (Supplement Fig. 16A–B; n = 3 NV samples per group, 157.0 [IQR 14.50] nm vs. 157.8 [IQR 33.90] nm, P > 0.05 by paired Wilcoxon test). The intrathecal NV and NV-miR injection had no change on the normal pain sensitive (Supplement Fig. 17A–E), motor function (Supplement Fig. 16F–G), DRG, and spinal cord morphology (Supplement Fig. 17H). The transfected NV-miR was subsequently delivered to the CCI model. There were no significant differences between the groups in maximum radiant efficiency (Supplement Fig. 18; n = 3 mice per group, F [1, 4] = 0.055, P = 0.8221 by two-way ANOVA) or diffusion distance (Fig. 1E; 1.13 ± 0.29 cm *vs*. 0.95 ± 0.38 cm, P = 0.7330 by unpaired t‐test). Treatment with NV-encapsulated transfected miR-101b-3p (NV-miR) significantly restored the mechanical threshold, with the analgesic effect lasting up to day 16 postsurgery (Fig. 7D; n = 10 mice per group, two-way RM ANOVA followed by Holm-Sidak *post hoc* test). The NV-miR group presented a significant reduction in response to the noxious pinprick test compared with the NV group (Fig. 7E; n = 10 mice per group, 1165 [IQR 185] ms *vs.* 735 [IQR 175] ms, P = 0.0303 by Kruskal-Wallis test followed by Dunn's *post hoc* test). Additionally, the CCI model exhibited hyposensitivity to heat and cold thermal stimulation after treatment with NV-miR (Fig. 7F and G; n = 10 mice per group, two-way RM ANOVA followed by Holm-Sidak *post hoc test*). These findings suggest that NV-encapsulated miR-101b-3p is a promising approach for enhancing analgesic effects in a preclinical model.

**Fig. 7:**
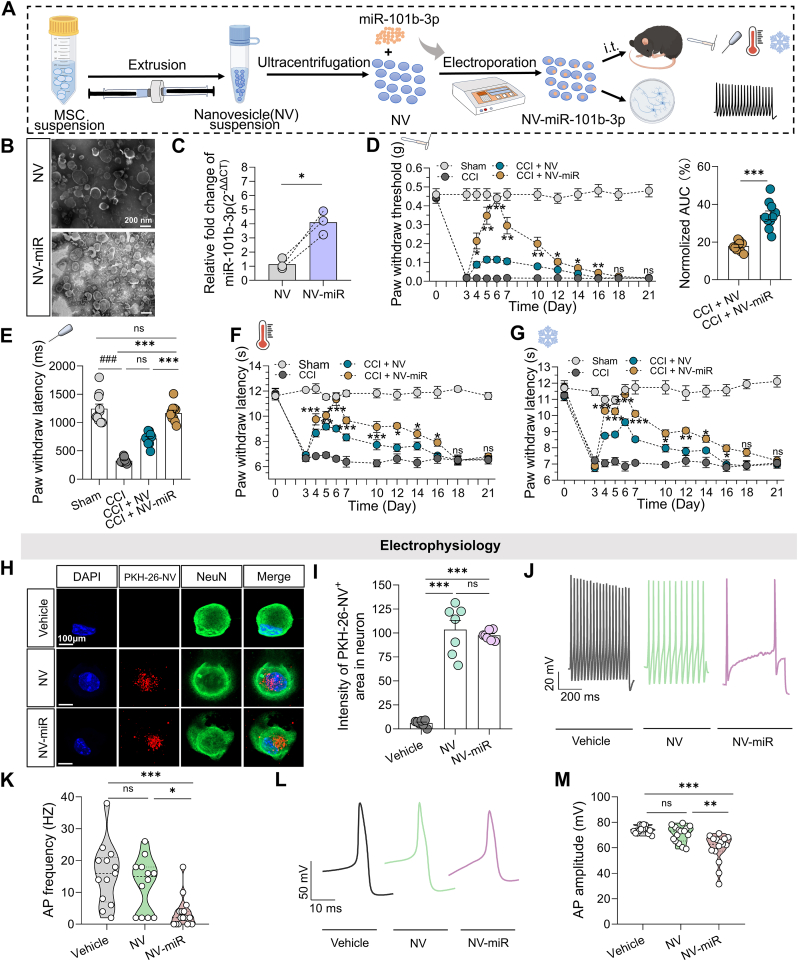
NV-encapsulated miR-101b-3p reduces pain **behavior.** (**A**) Schematic representation of the experimental process. (**B**) TEM showing the morphology of NV and NV-miR-101b-3p (scale bar, 200 nm). (**C**) Level of miR-101b-3p in NV before and after electroporation. (**D**) von-Frey test (left panel, n = 10 mice), normalized AUC (right panel). (**E**) Pinprick test (n = 10 mice). (**F**) Paw withdrawal latency of the Hargreaves test (n = 10 mice). (**G**) Latency to withdrawal of dry ice-induced cold stimulation (n = 10 mice). (**H-I**) Fluorescence images showing NV and NV-miR-101b-3p uptake by DRG neurons (n = 7 cells, scale bar, 100 μm). Red color indicates PKH-26-labeled NV or NV-miR, and green color indicates NeuN. (**J**) Sample of AP firing. (**K**) Summary data of AP frequency (n = 13–15 cells from 3 mice). (**L**) Sample of AP amplitude. (**M**) Summary data of AP amplitudes (n = 15 cells from 3 mice). TEM: Transmission electron microscope; NTA: nanoparticle tracking analysis; NV: Nanovesicle; AP: Action potential; DRG: Dorsal root ganglion; CCI: Chronic constriction injury model. All data are expressed as means ± SEM or median (IQR). ∗P < 0.05, ∗∗P < 0.01, ∗∗∗P < 0.001, ^###^P < 0.001, ns: no significant.

To investigate the effects of NV-encapsulated miR-101b-3p on neuronal function, we delivered NV-miR to DRG neurons isolated from a CCI model. Whole-cell patch-clamping recordings were subsequently performed to assess neuron activity. We confirmed that DRG neurons effectively internalized NV-miR (Fig. 7H–I; n = 7 cells per group). Notably, treatment with NV-miR resulted in a significant reduction in the AP firing rate compared with that of the NV group (Fig. 7J–K; n = 13–15 cells from 3 mice per group, P = 0.0274 by Kruskal-Wallis test followed by Dunn's *post hoc* analysis). NV-miR also significantly decreased the AP amplitude in the neurons (Fig. 7L–M; n = 15 cells from 3 mice, NV-miR *vs.* NV: 62.74 [IQR 9.56] mV *vs.* 73.12 [IQR 10.86] mV, P = 0.0061 by Kruskal-Wallis test followed by Dunn's *post hoc* analysis). These data demonstrate that NV-encapsulated miR-101b-3p effectively attenuates the hyperexcitability of neurons, suggesting its potential as an analgesic agent.

## 4 Discussion

Previous studies have shown that MSC-EV alleviates neuropathic pain [13,14]. This study demonstrated that compared with MCS-derived EV, EV derived from TNF-α-primed MSC had greater analgesic effects compared with MSC-derived EV in mechanical and thermal hypersensitivity. Notably, T-EV significantly improved the mechanical pain threshold, with effects persisting to day 18 post-CCI surgery. Additionally, T-EV reduced the hyperexcitability of DRG neurons. This study revealed elevated content of miR-101b-3p in T-EV by multiple approaches, and silencing miR-101b-3p in T-EV abrogated their enhanced analgesic effects. These findings demonstrated that miR-101b-3p, which was encapsulated within T-EV, acted as a crucial mediator of the enhanced analgesic effects of T-EV. Furthermore, miR-101b-3p appeared to exert its analgesic effects by targeting Nav1.6, which was overexpressed in DRG neurons in the neuropathic pain model. These results suggest that T-EV represents a promising therapeutic strategy for neuropathic pain, while replenishment of miR-101b-3p may serve as an effective approach to achieve similar analgesic benefits. Additionally, the delivery of miR-101b-3p *via* NV to treat neuropathic pain represents a feasible, practical, and long-term therapeutic approach.

In recent years, regenerative medicine has emerged as a promising approach for pain management, with preclinical and clinical studies highlighting the therapeutic potential of MSC-EV [44,45]. A single intrathecal injection of human MSC-derived EV has been shown to alleviate mechanical hypersensitivity in neuropathic pain models [13,46]. However, EV derived from MSC under resting conditions did not fully mitigate neuropathic pain following early intervention [13]. Furthermore, the analgesic effects and their duration have varied across studies, with some inconsistency in the outcomes [13,46,47]. To enhance the therapeutic efficacy of MSC-EV, several strategies, including both genetic and non-genetic approaches, have been explored. While some genetic modifications have shown promise in increasing the expression of specific proteins or microRNAs in MSC-EV, their clinical translation remains challenging. Given the inherent plasticity of MSC, the surrounding microenvironment plays a critical role in modulating their activity and function [20]. Preconditioning MSC with specific soluble molecules has thus become a well-established strategy for improving their therapeutic potential in regenerative medicine [22,23]. In our study, we have found that EV extracted from TNF-α-primed MSC enhanced the pain threshold in a neuropathic pain model and provided complete pain relief in the short term.

Previous studies have suggested that the analgesic effects of MSC-EV were primarily attributed to their well-established role in immune regulation [46]. However, our investigation revealed that treatment with T-EV did not significantly reduce inflammatory mediators compared with those in C-EV. Moreover, there is a lack of studies directly investigating their effects on sensory neurons under neuropathic pain conditions. In this study, we observed the direct action of EV from TNF-α-primed MSC on DRG neurons. Our results showed that T-EV restored normal firing rates and amplitudes of APs in DRG sensory neurons, thereby effectively attenuating their excessive excitability. These findings suggest that T-EV can directly modulate the hypersensitivity of sensory neurons, contributing to their analgesic effects in neuropathic pain.

At the same dose, no significant difference in the uptake of T-EV or C-EV by DRG neurons was observed. These findings suggest that the enhanced analgesic effect of T-EV is not attributable to the amount of T-EV taken up but rather may be linked to differences in their bioactive cargo. Moreover, the bioactive contents of MSC-EV are well-established as critical factors influencing their therapeutic efficacy [48]. Several miRNAs encapsulated in MSC-EV, such as miR-26a-5p, have been identified as potential regulators of pain relief mechanisms [14,46]. However, the precise role of these miRNAs in pain modulation remains unverified, primarily owing to insufficient experimental validation. Moreover, while the involvement of miRNAs in pain processing has been suggested, the underlying mechanisms by which they influence pain thresholds are not yet fully understood. In this study, we investigated the differential expression of miRNAs in C-EV and T-EV, and identified miR-101b-3p as a critical mediator of the enhanced analgesic effects of T-EV. Specifically, miR-101b-3p was found to be downregulated in the DRG after CCI surgery. Silencing miR-101b-3p expression in T-EV attenuated its analgesic effect on nociceptive behaviors, and overexpression of miR-101b-3p in NV further potentiated pain relief. However, miR-101b-3p did not significantly contribute to the attenuation of the brush response following T-EV treatment, which represents a form of light mechanical stimulation. This effect may be attributed to the distinct functional roles of Nav1.6 across different DRG neuronal subtypes, which may contribute to the different types of sensory transmission. The complexity of the EV content and the fact that the basic analgesic effects of T-EV were not fully abolished by silencing miR-101b-3p suggest the participation of other potential molecular factors in mediating its therapeutic effects. The other miRNAs may also be a molecule of analgesic effect in T-EV. We have also provided additional differential protein omics data in Supplementary Fig. 2 to facilitate further investigation for future research.

MiRNAs are well known for their ability to regulate multiple genes and cellular pathways, which makes them promising therapeutic candidates for the treatment of neuropathic pain [49]. For example, miR-32–5p has been shown to target Cav3.2 channels, alleviating CCI-induced pain [50], while the miR-183 cluster regulated over 80 % of genes associated with neuropathic pain, including those involved in voltage-gated calcium channels, thereby reducing mechanical pain sensitivity [51]. Additionally, the miR-30 family has been implicated in regulating Nav1.7 expression in the human epidermis [52]. These findings highlight the critical role of miRNAs in modulating ion channel genes and their potential to promote pain relief in neuropathic disorders [53]. Although the intrathecal injection of miR-101b-3p had a direct analgesic effect, this effect did not persist over time. In contrast, both T-EV and NV-loaded miRNA provided more sustained pain relief, with effects more than 10 days post-CCI surgery. We speculate that the lipid bilayer of EV and NV plays a key role in the controlled release of bioactive molecules, thereby prolonging the duration of the analgesic effect. Moreover, NV-encapsulated miR-101b-3p offered both enhanced therapeutic efficacy and a straightforward approach for potential clinical application.

Our results indicate that TNF-α plays a critical role in modulating the loading efficiency of miRNAs into EV derived from parent cells. Previous studies have demonstrated that TNF-α acted as an activator, upregulating the expression of various biomolecules through the nuclear factor-κB (NF-κB) and MAPK signaling pathways, which subsequently enhances the secretion of EV-associated proteins in MSC [21,22]. The result has shown NF-κB pathway also regulates the upregulation of miR-101b-3p in EV after TNF-α stimulation. However, the precise mechanisms by which TNF-α regulates miRNA loading into EV or promotes miRNA biogenesis remain to be fully elucidated.

MiRNAs regulate target gene expression by interacting with the Argonaute (AGO) protein, which binds to regions of imperfect complementarity within the 3′ UTR of target mRNAs. This interaction leads to mRNA degradation and a reduction in protein translation [54]. In several gene-target databases, VGSCs were identified as potential targets of miR-101b-3p. We also observed that the AP amplitude was restored following T-EV treatment. After the knockdown of miR-101b-3p in the T-EV, in addition to excessive excitability showing no changes, the AP amplitude failed to be reversed, suggesting that VGSCs played a role in this regulation. Over the past two decades, significant efforts have been directed at developing novel analgesics targeting VGSCs expressed in nociceptive neurons [[55], [56], [57]]. Our study focused on key VGSCs involved in the regulation of sensory neuron activity, including Nav1.6, Nav1.7, and Nav1.8. Notably, we found that Nav1.6 expression was increased following neuropathic pain modeling. In a spinal nerve ligation (SNL) model, local knockdown of Nav1.6 significantly reduced mechanical pain behaviors and suppressed abnormal spontaneous activity and hyperexcitability in the ligated sensory ganglion [58,59]. Furthermore, Nav1.6 has been implicated in the functional alterations of DRG neurons following vincristine treatment, where it plays a crucial role in maintaining neuropathic allodynia [27]. Interestingly, microenvironmental TNF-α in the nervous system was shown to upregulate Nav1.6 expression, increasing neuronal excitability [[60], [61], [62]]. In this study, we demonstrated that miR-101b-3p, which was enriched in EV derived from TNF-α-primed MSC, effectively attenuated the upregulation of Nav1.6 expression in a neuropathic pain model. It has been reported that Nav1.6 significantly contributes to the rising phase of the AP and the rapid activation kinetics of channel [55]. The dual luciferase reporter gene assay confirmed that Nav1.6 was the direct target of miR-101b-3p. MiR-101b-3p notably reduced both the firing rate and the AP amplitude in sensory neurons, suggesting that miR-101b-3p modulates neuronal excitability by inhibiting Nav1.6 expression potentially.

There are some limitations in the study. We implemented early intervention in the CCI model to investigate the analgesic effect. The CCI model was employed in this study due to its technical accessibility, high reproducibility, and rapid induction of measurable pain hypersensitivity. Crucially, this model recapitulates dual neuropathic-inflammatory pathology, providing a clinically relevant representation of post-traumatic peripheral neuropathy. However, neuropathic pain in humans is typically more persistent and complex, necessitating further investigation to enhance translational relevance. Moreover, while our study focused on the peripheral nervous system, the potential involvement of the central nervous system in the analgesic effects of EV warrants further investigation. The clinical application of EV for pain management remains challenging. Although we observed elevated levels of miR-101b-3p in T-EV derived from human BMSC, supporting the translational potential of our findings, the large-scale production and stringent quality control required for clinical-grade EV continue to present significant obstacles. Moreover, Nav1.6 has been identified as a direct target of miR-101b-3p in neurons, but miRNA regulates multiple targets, thereby influencing a wide range of biological processes [63,64]. The genome encodes hundreds of conserved miRNAs, each capable of modulating numerous target mRNAs [54]. Previous studies have suggested that miR-101b-3p regulates sevoflurane-induced memory and learning impairments by targeting receptor signaling pathways in the hippocampus [65]. MiR-101b-3p may also interact with other neural targets implicated in pain processing.

In summary, TNF-α-stimulated MSC-derived EV enhanced analgesic effects in neuropathic pain by directly modulating the excessive excitability of sensory neurons. A critical mediator of pain relief is miR-101b-3p, which directly targets Nav1.6 in sensory neurons, thereby influencing neuronal excitability. While these findings provide promising insights into the therapeutic potential of EV-based treatments, further extensive preclinical studies are essential to validate the efficacy, safety, and mechanisms underlying EV-mediated pain relief before their clinical translation.

## CRediT authorship contribution statement

**Lanyu Zhang:** Writing – original draft, Writing – review & editing, Validation, Software, Resources, Project administration, Methodology, Investigation, Funding acquisition, Formal analysis, Data curation, Conceptualization. **Jinping Wang:** Writing – review & editing, Validation, Project administration, Methodology, Investigation, Formal analysis, Data curation. **Jin Liu:** Writing – review & editing, Validation, Supervision, Resources, Data curation. **Juan Xin:** Project administration, Methodology, Investigation, Funding acquisition, Formal analysis, Writing – review & editing. **Yuan Tan:** Methodology, Investigation, Data curation. **Donghang Zhang:** Writing – review & editing, Visualization, Validation, Supervision. **Tao Zhu:** Validation, Funding acquisition, Data curation, Writing – original draft, Writing – review & editing, Supervision, Conceptualization. **Cheng Zhou:** Conceptualization, Funding acquisition, Writing – review & editing, Writing – original draft, Validation, Supervision, Resources, Data curation.

## Availability of data and materials

All data analyzed during this study are included in this published article (and its supplementary information files).

## Ethics approval and consent to participate

The study was approved by the Experimental Animal Ethics Committee of West China Hospital, Sichuan University (approval number 20221008006).

## Funding

This work was supported by the 10.13039/501100001809National Natural Science Foundation of China (Grant No. 82371281 to T.Z., No. 82271290 to C.Z., No. 82201341 to J.X. and No.82301360 to J.W.), the 10.13039/501100004829Sichuan Provincial Department of Science and Technology (Grant No. 2023ZYD0168 to C.Z.), and the 10.13039/501100018542Natural Science Foundation of Sichuan Province (Grant No. 2024NSFSC1635 to L.Z.).

## Declaration of competing interest

The authors declare that they have no known competing financial interests or personal relationships that could have appeared to influence the work reported in this paper.

## Figures and Tables

**figs1:**
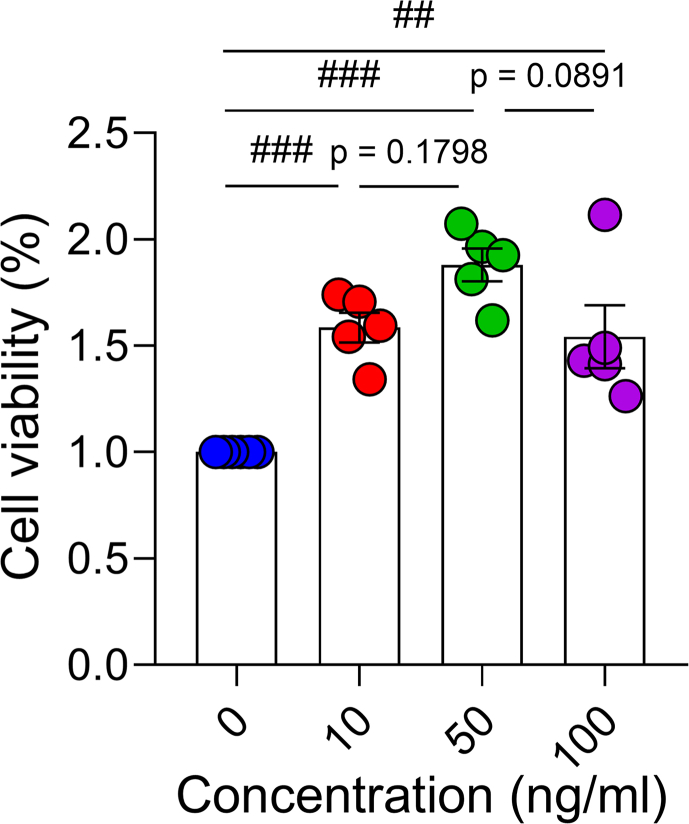
**MSC viability by TNF-α** Cell viability of MSC after different concentrations of TNF-α stimulation (n = 5 dishes). MSC: Mesenchymal stem cell. All data are expressed as means ± SEM. ^##^ P < 0.01, ^###^ P < 0.001, ns: no significant.

**figs2:**
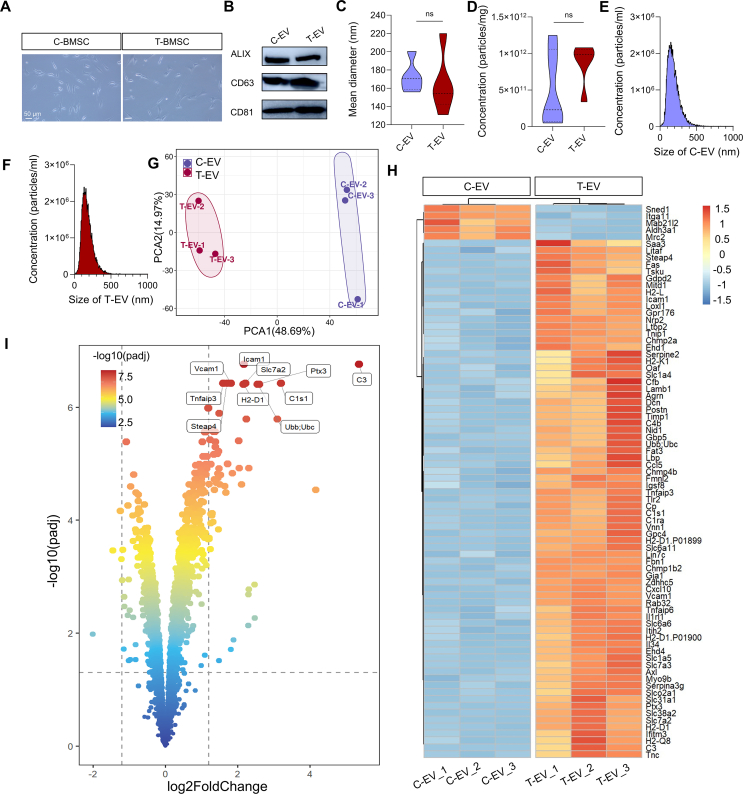
**Characteristics of C-EV and T-EV** (**A**) Morphology of MSC after TNFα stimulation (scale bar, 50 μm). (**B**) The expression of protein makers on C-EV and T-EV. (**C**) Mean diameter of C-EV and T-EV by NTA (n = 5 EV samples). (**D**) The concentration of EV (n = 5 EV samples). (**E**) The size distribution of C-EV. (**F**) The size distribution of T-EV. (**G**) PCA analysis of C-EV and T-EV (n = 3 EV samples). (**H**) Heatmap of the differential proteins between C-EV and T-EV. (**I**) Volcano of the differential proteins between C-EV and T-EV. C-BMSC: Bone marrow-derived MSC; T-BMSC: TNF-α-preconditioned bone marrow-derived MSC; C-EV: Control mesenchymal stem cell-derived extracellular vesicle; T-EV: TNF-α-preconditioned mesenchymal stem cell-derived extracellular vesicle. PCA: Principal Component Analysis. All data are expressed as median (IQR). ns: no significant.

**figs3:**
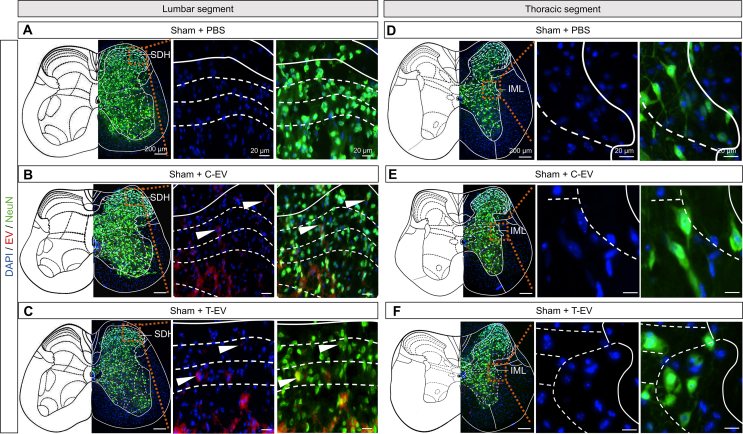
**Distribution of EV in spinal dorsal horn and intermediolateral nucleus** (**A**) The distribution of PBS solution in SDH (scale bar, left, 200 μm, right, 20 μm). (**B**) The distribution of PKH-26-labeled C-EV in SDH. (**C**) The distribution of PKH-26-labeled T-EV in SDH. (**D**) The distribution of PBS solution in IML (scale bar, left, 200 μm; right, 20 μm). (**E**) The distribution of PKH-26-labeled C-EV in IML. (**F**) The distribution of PKH-26-labeled T-EV in IML. The white arrow indicates PKH-26-labeled EV; red color represents PKH-26-labeled EV, and green color indicates NeuN. EV: Extracellular vesicle. SDH: Spinal dorsal horn; IML: Intermediolateral nucleus.

**figs4:**
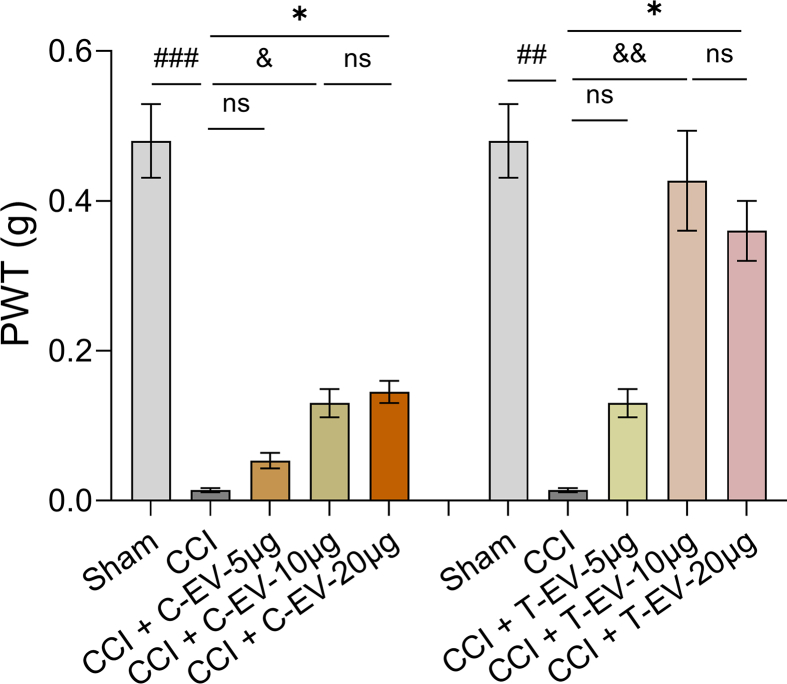
**Different dosages of EV for analgesic effect.** (**A**) Paw withdrawal threshold after von Frey test following intrathecal delivery of 5 μg, 10 μg, and 20 μg of C-EV or T-EV (n = 5-6 mice per group). C-EV: Control mesenchymal stem cell-derived extracellular vesicle; T-EV: TNF-α-preconditioned mesenchymal stem cell-derived extracellular vesicle; CCI: chronic constriction injury. All data are expressed as means ± SEM. ^#^ P < 0.05, ^##^ P < 0.01, ^&^ P < 0.05, ^&&^ P < 0.01, ^∗^P < 0.05, ns: no significant.

**figs5:**
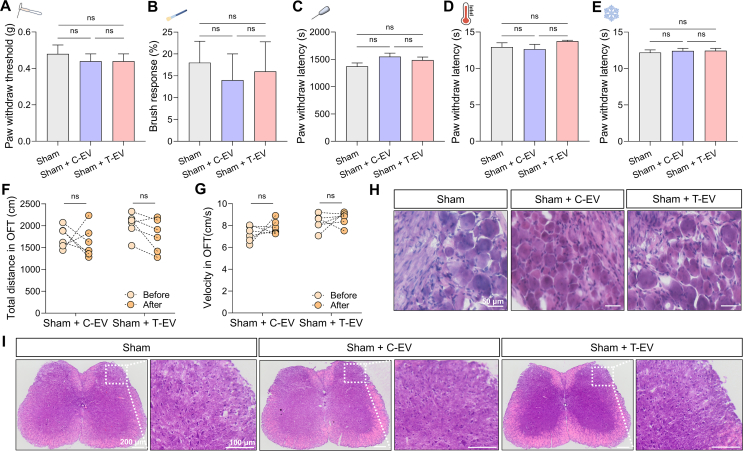
**Behavioral, locomotor, and histological assessments following intrathecal administration of EV in *naïve* mice** (**A**) Paw withdrawal threshold after von-Frey test (n = 5 mice). (**B**) Brush response test (n = 5 mice). (**C**) Response time of pinprick test (n = 5 mice). (**D**) Latency to withdrawal after Hargreaves test (n = 5 mice). (**E**) Latency to withdrawal after ice-cold stimulation (n = 5 mice per group). (**F**) Total distance of mice before and after intrathecally delivering C-EV or T-EV (n = 5 mice). (**G**) Velocity of mice before and after intrathecally delivering C-EV or T-EV. (**H**) HE staining of DRG after intrathecal delivery of EV (scale bar, 50 μm). (I) HE staining of the spinal cord after intrathecal delivery of EV (scale bar, left, 200 μm; right, 100 μm). C-EV: Control mesenchymal stem cell-derived extracellular vesicle; T-EV: TNF-α-preconditioned mesenchymal stem cell-derived extracellular vesicle; HE: Hematoxylin-eosin. All data are expressed as means ± SEM. ns: no significant.

**figs6:**
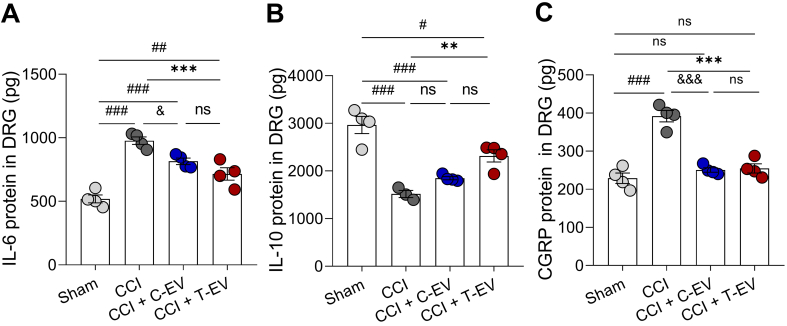
**Protein expression of inflammatory mediators by ELISA.** (**A**) Expression of IL-6 protein in DRG on Day 6 (n = 4 mice). (**B**) Expression of IL-10 protein in DRG on Day 6 (n = 3-4 mice). (**C**) Expression of IL-10 protein in DRG on Day 6 (n = 4 mice). All data are expressed as means ± SEM. ^#^ P < 0.01, ^##^ P < 0.

**figs7:**
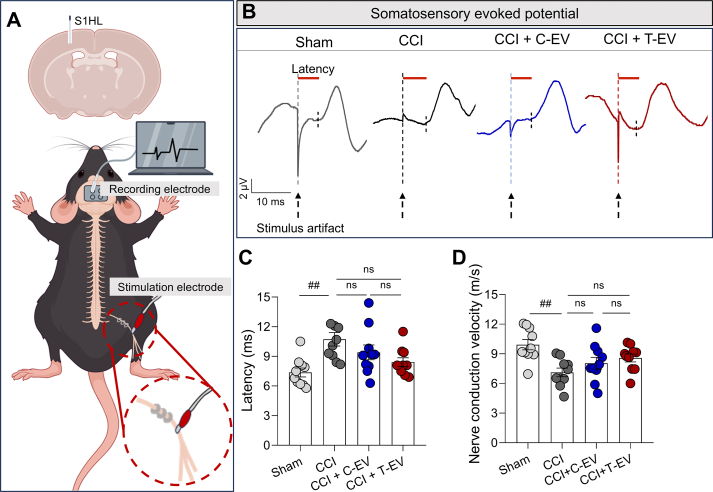
**T-EV did not affect sciatic nerve conduction velocity** (A) Diagram of the experiment process for recording SEP. (B) Representative samples of somatosensory evoked potential across groups. The black arrow indicates the stimulus artifact. (C) Latency recording of CCI with stimulated ipsilesional sciatic nerves. (D) Conduction velocity recording of CCI with stimulated ipsilesional sciatic nerves. SEP: Somatosensory cortical evoked potential; CCI: Chronic constriction injury. ^#^ P < 0.05, ^##^ P < 0.01, ns: no significant.

**figs8:**
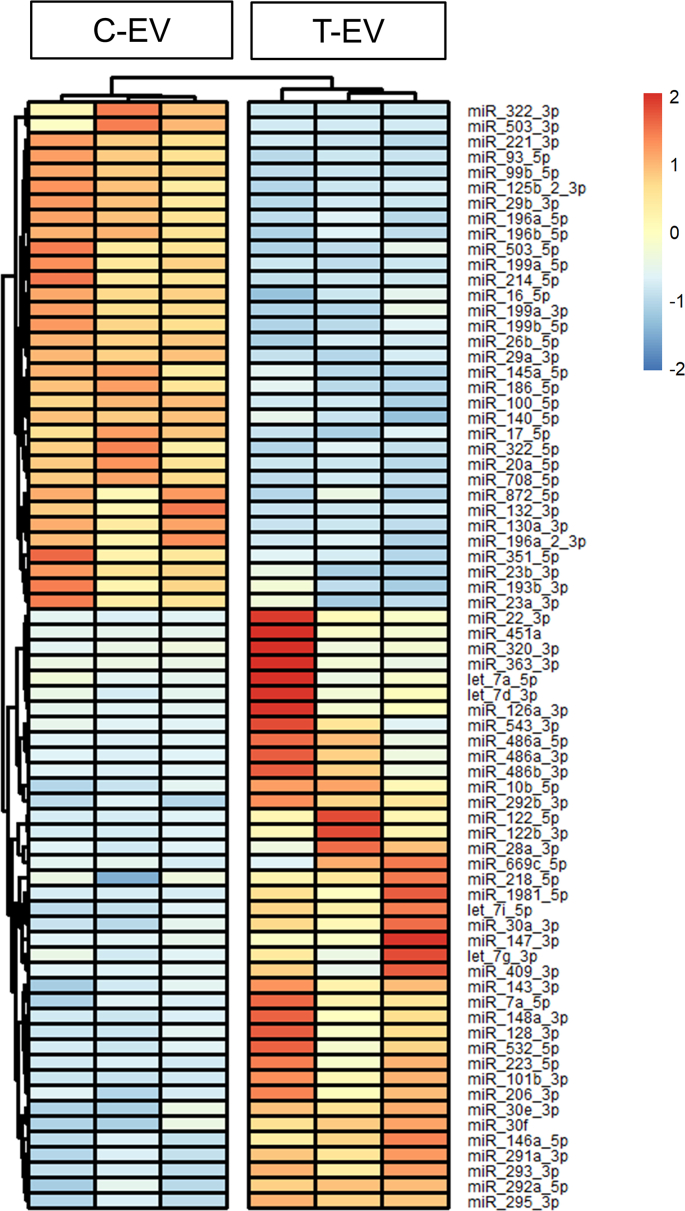
**Differential miRNAs by RNA-seq** All differential miRNAs between C-EV and T-EV in a heatmap. C-EV: Control mesenchymal stem cell-derived extracellular vesicle; T-EV: TNF-α-preconditioned mesenchymal stem cell-derived extracellular vesicle.

**figs9:**
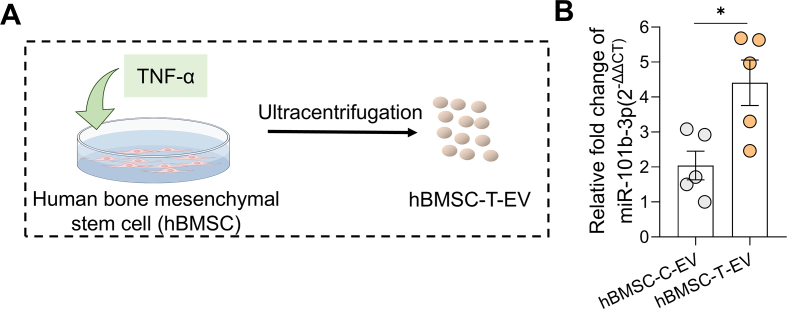
**TNF-α enhanced expression of miR-101b-3p in hBMSC-EV** (**A**) Diagram of experiment process. (**B**) miR-101b-3p expression in hBMSC-C-EV and hBMSC-T-EV (n = 5 EV samples). ^∗^ P < 0.05, ns: no significant.

**figs10:**
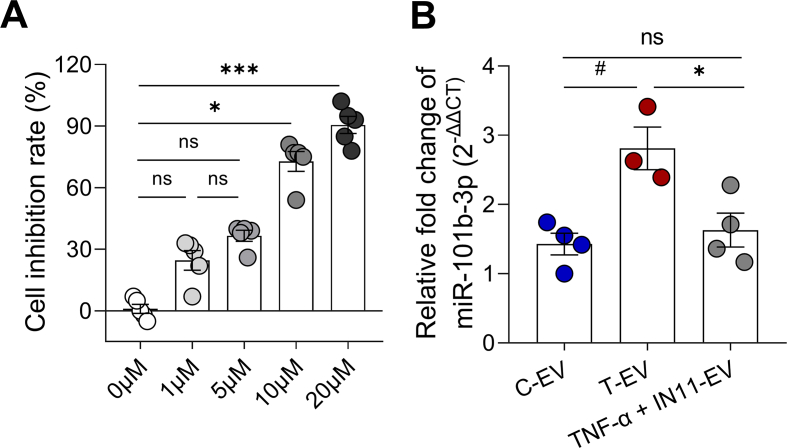
**NF-κB inhibitor inhibited the expression of miR-101b-3p in T-EV**(**A**) Different doses of IN-11 were used to assess the inhibition rate on MSC. (**B**) Expression of miR-101b-3p in EV after IN-11 treatment (n = 3-4 EV samples). ^#^ P < 0.05, ^∗^ P < 0.05, ^∗∗∗^ P < 0.001, ns: no significant.

**figs11:**
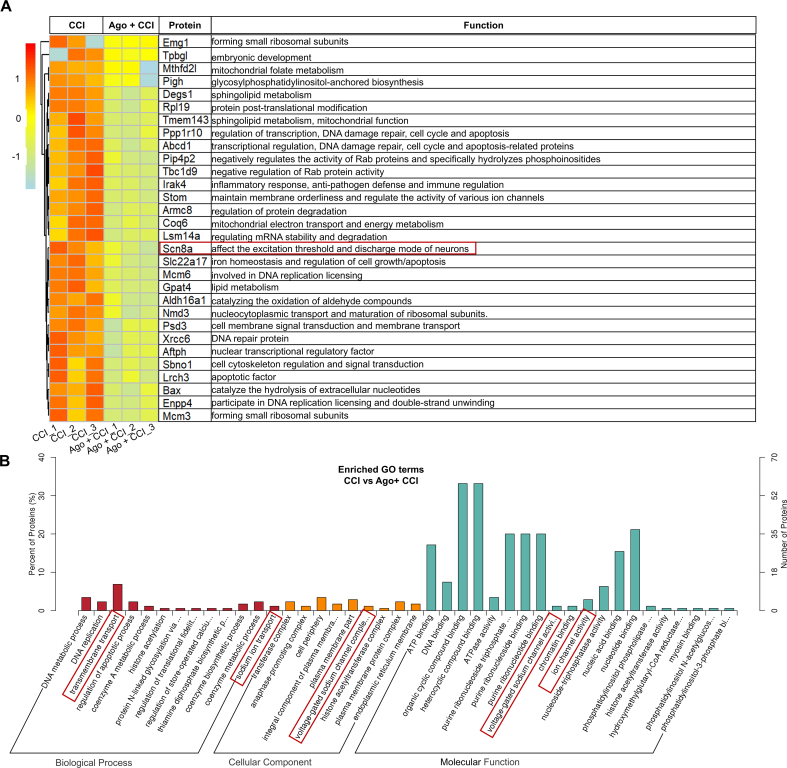
**Proteomic analysis of DRG** (**A**) Heatmap and function of differential proteins after DRG protein sequencing (n = 3 DRG). (**B**) GO analysis of differential proteins. DRG: dorsal root ganglion; GO: Gene ontology.

**figs12:**
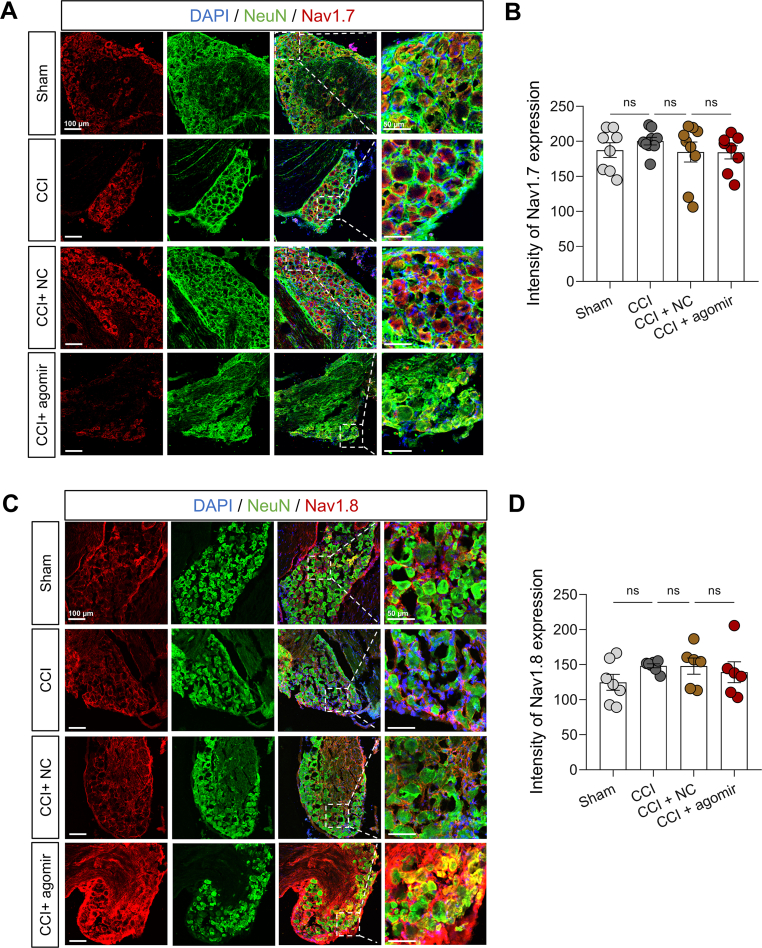
**Effect of miR-101b-3p on the expression of Nav1.7 and Nav1.8 on DRG** (**A**)The expression of Nav1.7 on DRG after intrathecal delivery of miR-101b-3p agomir by immunofluorescence (scale bar, left, 100 μm; right, 50μm). Red color indicates Nav1.7 expression, and green color indicates NeuN expression. (**B**) Semiquantitative analysis of DRG by immunofluorescence for Nav1.7 (n = 8-9 slices from 3 mice). (**C**) The expression of Nav1.8 on DRG after intrathecal delivery of miR-101b-3p agomir by immunofluorescence (scale bar, left, 100 μm; right, 50 μm). Red color indicates Nav1.8 expression, and green color indicates NeuN expression. (**D**) Semiquantitative analysis of DRG by immunofluorescence for Nav1.8 (n = 6-7 slices from 3 mice). CCI: chronic constriction injury. All data are expressed as means ± SEM. ns: no significant.

**figs13:**
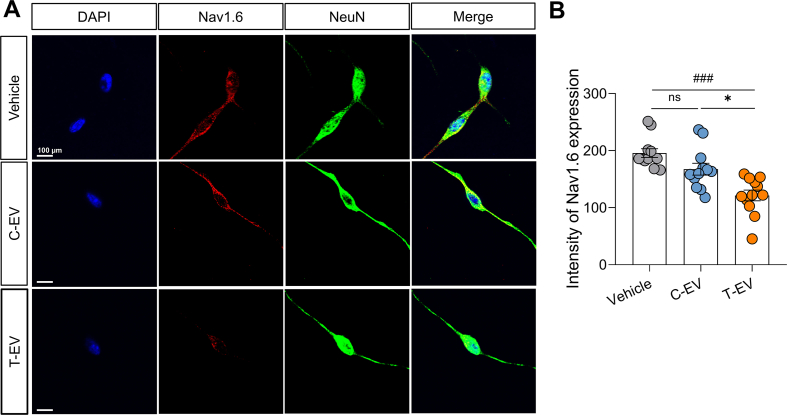
**The effect of EV on Nav1.6 expression of DRG neurons** (**A**) Fluorescence image showing the expression of Nav1.6 on DRG primary neurons from the CCI model following EV incubation (scale bar, 100 μm). Red color indicates Nav1.6 expression, and green color indicates NeuN expression. (**B**) Semiquantitative analysis of Nav1.6 expression by immunofluorescence for Nav1.6 (n = 12 cells). CCI: Chronic constriction injury. C-EV: Control extracellular vesicle; T-EV: TNF-α-preconditioned mesenchymal stem cell-derived extracellular vesicle; CCI: chronic constriction injury. All data are expressed as means ± SEM. ^∗^ P < 0.05, ^###^ P < 0.001. ns: no significant.

**figs14:**
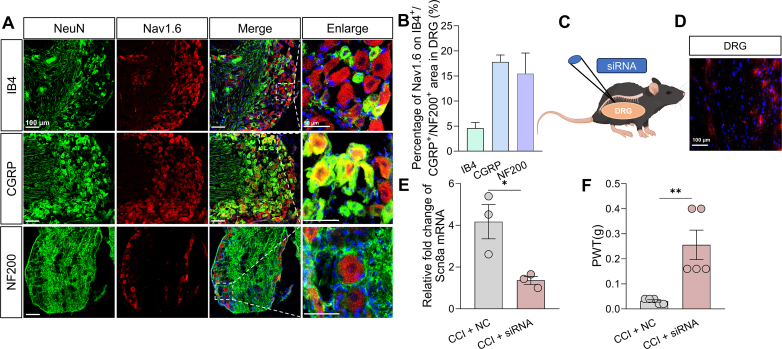
**Silencing Nav1.6 has an analgesic effect** (**A**) Nav1.6 is expressed in IB4^+^, NF200^+,^ and CGRP^+^ neurons (scale bar, left, 100 μm, right, 50 μm). (**B**) The expression percentage of Nav1.6 on IB4^+^, CGRP^+^, and NF200^+^ neurons (n = 4-5 slices from 4-5 mice). (**C**) Schematic of siRNA delivery to DRG. (**D**) Fluorescence imaging of DRG after siRNA injection (scale bar, 100 μm). (**E**) Scn8a mRNA expression in DRG after siRNA injection by RT-qPCR (n = 4 DRG samples in sham group, n = 3 DRG samples in CCI group). (**F**) Von-Frey test after knockdown of Nav1.6 (n = 4 mice in sham group, n = 5 mice in CCI group). CCI: chronic constriction injury. All data are expressed as means ± SEM. ^∗^ P < 0.05, ^∗∗^ P < 0.01, ns: no significant.

**figs15:**
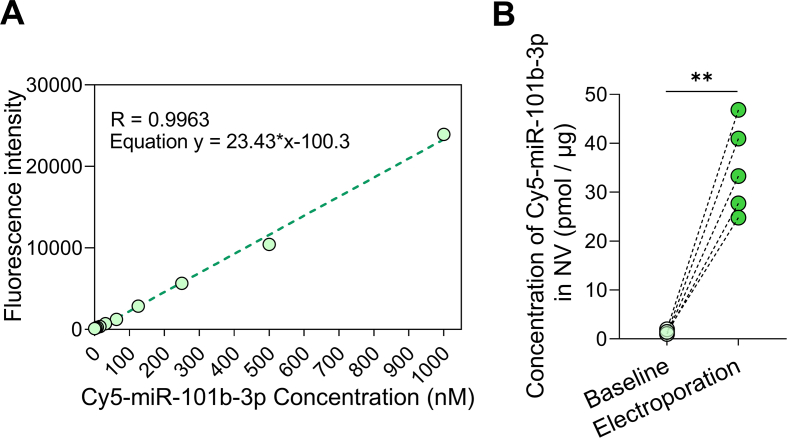
**Cy5-miR-101b-3p concentration in NV by electroporation** (**A**) Standard curve of Cy5-miR-101b-3p concentration and fluorescence intensity. (**B**) The Cy5-miR-101b-3p concentration in NV before and after electroporation. Cy5-miR-101b-3p: Cy5-labeled miR-101b-3p. ^∗∗^ P < 0.01.

**figs16:**
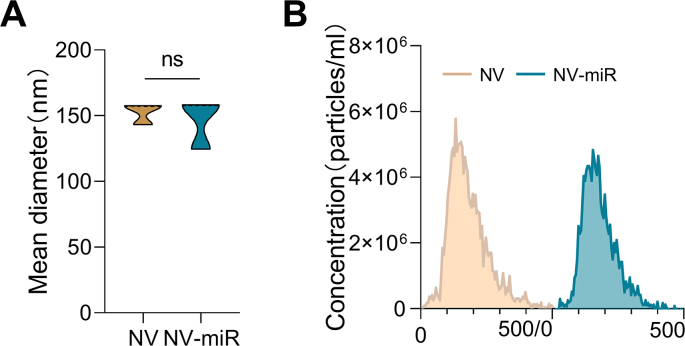
**Characteristics of NV and NV-miR** (**A**) Mean size diameter of NV and NV-miR. (**B**) Size distribution of NV and NV-miR (n = 3 NV samples). NV: nanovesicle; NV-miR: NV-encapsulated transfected miR-101b-3p. All data are expressed as means (IQR). ns: no significant.

**figs17:**
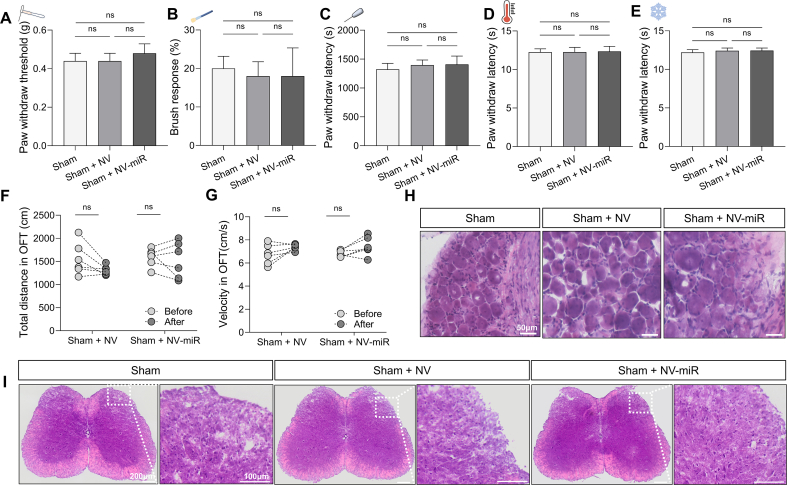
**Behavioral, locomotor, and histological assessments following intrathecal administration of NV in *naïve* mice** (**A**) Paw withdrawal threshold after von-Frey test (n = 5 mice). (**B**) Brush response test (n = 5 mice). (**C**) Response time of the pinprick test (n = 5 mice). (**D**) Latency to withdrawal after Hargreaves test (n = 5 mice). (**E**) Latency to withdrawal after dry ice-cold stimulation (n = 5 mice). (**F**) Total distance of mice before and after intrathecally delivering NV or NV-miR (n = 5 mice). (**G**) Motor velocity of mice before and after intrathecally delivering NV or NV-miR. (**H**) HE staining of DRG after intrathecal delivery of NV and NV-miR (scale bar, 50 μm). (I) HE staining of the spinal cord after intrathecal delivery of NV and NV-miR (scale bar, left, 100 μm; right, 50 μm). HE: Hematoxylin-eosin. NV: nanovesicle; NV-miR: NV-encapsulated transfected miR-101b-3p. All data are expressed as means ± SEM. ns: no significant.

**figs18:**
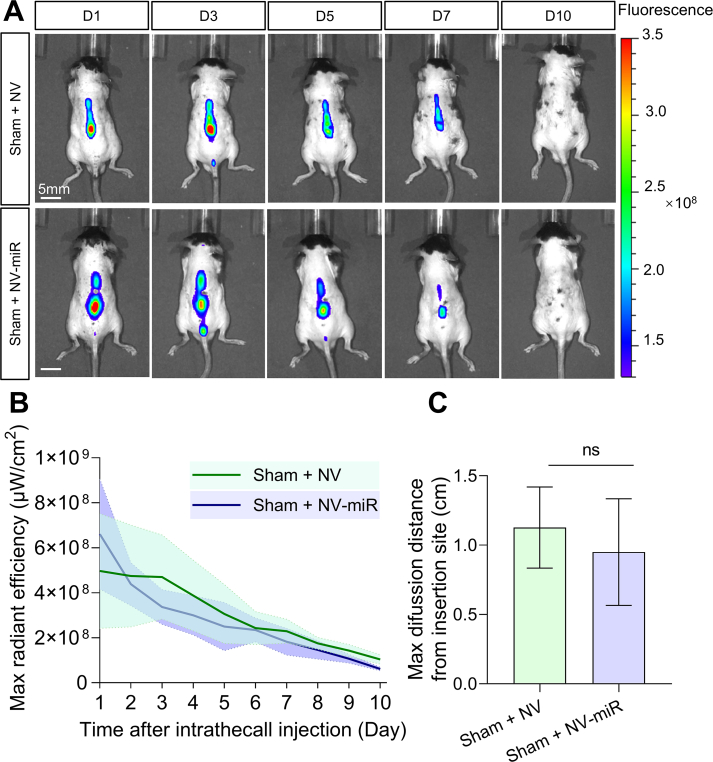
***In vivo* tracing of NV and NV-miR** (**A**) DiR-labeled NV and NV-miR tracing by IVIS system (scale bar = 5 mm). (**B**) The maximum radiant efficiency of intrathecal DiR-NV and DiR-NV-miR. (**C**) Maximum diffusion distance of DiR-labeled NV or NV-miR from the injection site on Day 2. NV: nanovesicle; NV-miR: NV-encapsulated transfected miR-101b-3p; IVIS: *in vivo* tracing. ns: no significant.

**figs19:**
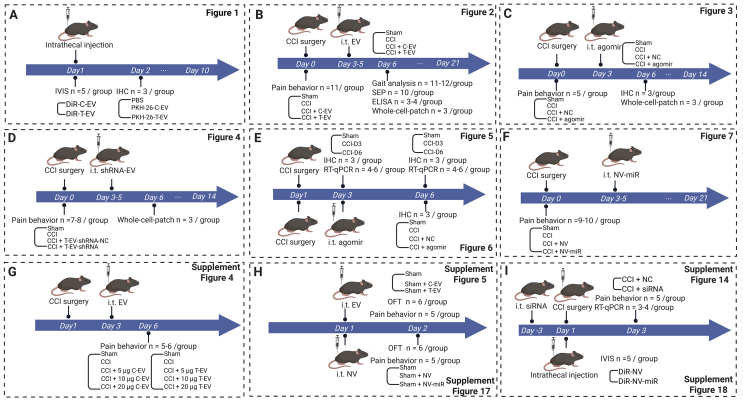
**Overview of the main experimental procedures** (**A**) Experimental design and workflow for Figure 1C-H. (**B**) Experimental design and workflow for Figure 2B-R. (**C**) Experimental design and workflow for Figures 3E-O and 6A-B. (**D**) Experimental design and workflow for Figure 4F-P. (**E**) Experimental design and workflow for Figure 5E-J. (**F**) Experimental design and workflow for Figure 7D-H. (**G**) Experimental design and workflow for Supplementary Figure 4. (**H**) Experimental design and workflow for Supplementary Figures 5A-G and 17A-G. (**I**) Experimental design and workflow for Supplementary Figures 14C-F and 18A-C. CCI: Chronic constriction injury; IVIS: In Vivo Imaging System; T-EV: TNF-α-preconditioned mesenchymal stem cell-derived extracellular vesicle; C-EV: Control mesenchymal stem cell-derived extracellular vesicle; IHC: Immunohistochemistry; NV: Nanovesicle; OFT: Open field test; NC: Negative control.

## Abbreviations

MSC: Mesenchymal stem cell
EV: Extracellular vesicle
T-EV: TNF-α-preconditioned mesenchymal stem cell-derived extracellular vesicle
C-EV: Control mesenchymal stem cell-derived extracellular vesicle
IVIS: In Vivo Imaging System
DRG: Dorsal root ganglion
miRNA: microRNA
CCI: Chronic constriction injury
SPF: Specific pathogen-free
ELISA: Enzyme-linked immunosorbent assay
NV: Nanovesicle
ANOVA: Analysis of variance
RM ANOVA: repeated measures analysis of variance
TNF-α: Tumor necrosis factor-α
IL-1β: Interleukin-1β
IL-6: Interleukin-6
IL-10: Interleukin-10
CGRP: Calcitonin gene-related peptide
AP: action potential
AGO: Argonaute
3′ UTR: 3′ untranslated region
VGSC: voltage-gated sodium channel
SNL: spinal nerve ligation
IVIS: *in vivo* tracing system
PWT: paw withdrawal threshold
PWL: paw withdrawal latency
OFT: Open field test
SEP: Somatosensory evoked potential
hBMSC: Human bone-derived Mesenchymal stem cell
NC: Negative control
