# Supplementary material for: TNF-α-preconditioning enhances analgesic efficacy of mesenchymal stem cell-derived extracellular vesicle in neuropathic pain via miR-101b-3p targeting Nav1.6
Source: Bioact Mater. 2025 Jul 26;53:522–39. doi: 10.1016/j.bioactmat.2025.07.029 (PMC12313965; doi:10.1016/j.bioactmat.2025.07.029)
Supplement: Multimedia component 1 [file mmc1.docx]

**Supplement methods**

**Cell viability/inhibition detection by CCK-8**

Mesenchymal stem cell (MSC) was seeded in a 96-well plate and incubated overnight. The culture medium was then replaced with Dulbecco's Modified Eagle's Medium/Nutrient Mixture F-12 (DMEM/F12) (Gibco^TM^, USA) containing 0 ng/ml, 10 ng/ml, 50 ng/ml, or 100 ng/ml tumor necrosis factor-alpha (TNF-α), respectively, and the cells were cultured for 48 hours. Another 96-well plate was incubated with N-11, the NF-κB inhibitor (MCE, USA), at 0 nM, 5 nM, 10 nM, and 20 nM, respectively. Following this, CCK-8 reagent (APExBIO, USA) was added to these culture media, and the cells were incubated for another 4 hours. Cell viability and cell inhibition rate were assessed by measuring absorbance at 450 nm using a microplate spectrophotometer (EON, BioTek, USA). The resulting absorbance data were used to calculate cell viability or cell inhibition rate.

**Nanoparticle tracking analysis**

The size and concentration of EV were determined by nanoparticle tracking analysis (NTA) using a Zetaview instrument (Particle Metrix, Germany). Extracellular vesicle (EV) and Nanovesicle (NV) samples were diluted with PBS (1:1000 - 1:2000) and then injected into the instrument with a 1 ml syringe. NTA measurements were recorded and analyzed at 8-10 distinct positions to ensure accuracy and reliability. Then the data was analyzed by the Zetaview software (ZETAVIEW, Germany).

**Western blot**

EV was collected and isolated using RIPA lysis buffer (Thermo Fisher Scientific, USA). Protein concentrations were determined using a BCA protein assay (#23227, Thermo Fisher Scientific, USA). Equal amounts of protein (20 µg) were mixed with 4 × loading buffer, separated by SDS-PAGE on a 12.5% polyacrylamide gel (Epizyme, China), and subsequently transferred to a polyvinylidene difluoride (PVDF) membrane. The membrane was incubated with PBS containing 5% skim milk to block non-specific binding. Following blocking, the membrane was incubated overnight at 4°C with primary antibodies CD81 (Santa Cruz, USA), CD63 (Abcam, USA), and ALIX (Proteintech, China). The membrane was incubated with horseradish peroxidase (HRP)-conjugated secondary IgG (ZSGB-BIO, China) at room temperature for 1.5 hours the next day. The membrane was then tested by an enhanced chemiluminescence (ECL) detection kit (Bio-Rad, USA). Imaging was performed using an ultrasensitive multifunctional imaging system (AI600, GE, USA).

**Proteomic analysis**
Proteins were extracted from EV samples, and their concentrations were determined using a BCA assay. An integrated microcolumn containing a top-layer mixed SCX/SAX resin and a bottom-layer C18 membrane was employed to perform protein enrichment, reduction, alkylation, enzymatic digestion, and peptide desalting in a single workflow, followed by elution and lyophilization. The dried peptides were reconstituted in 0.1% formic acid, centrifuged, and aliquots were injected for analysis using a Vanquish Neo nano-UHPLC system coupled to a Thermo Orbitrap Astral mass spectrometer operated in DIA mode. Bioinformatics tools are then applied to process the raw data, identify differentially expressed proteins. Statistical analysis was performed in Perseus, and differentially abundant proteins between groups were defined by | log2(fold change) | ≥ 1.2 and P < 0.05.

**Sicatic nerve conduction velocity test *in vivo***

A recording electrode was stereotaxically implanted into the primary somatosensory cortex trunk region (S1HL, AP -1.00 mm, ML -1.67 mm, DV -1.00 mm) in mice, while a reference electrode was positioned in the vestibular nucleus (V1B, AP -3.80 mm, ML -2.50 mm, DV -1.00 mm). The stimulating electrodes were placed at the distal end of the sciatic nerve and delivered at 3 mA current using a Multi-channel Physiological Data Acquisition and Analysis System (RM6240XC, Chengdu Instrument Factory, Chengdu, China). The evoked potentials were recorded in S1HL. Nerve conduction velocity was calculated based on the latency of the recorded potential and the distance between the stimulation and recording sites.

**Immunofluorescence staining**

DRG slices were incubated with primary antibodies targeting specific markers. The primary antibody combinations included: Nav1.6 (Rabbit, 1:1000, Abcam) with NeuN (Mouse, 1:500, Abcam), Nav1.7 (Rabbit, 1:500, Bioss) with NeuN (Mouse, 1:500, Abcam), Nav1.8 (Mouse, 1:1000, Abcam) with NeuN (Rabbit, 1:500, Oasis Biofarm), CGRP (Goat, 1:1000, Abcam) with Nav1.6 (Rabbit, 1:1000, Abcam), and NF200 (Mouse, 1:800, Millipore) with Nav1.6 (Rabbit, 1:1000, Abcam), and Nav1.6 (Rabbit, 1:1000, Abcam) alone. All slices were incubated overnight at 4°C. After primary antibody incubation, DRG slices only incubated with Nav1.6 antibody were treated with IB4-FITC-conjugated 488 antibodies in combination with Cy™3-conjugated goat anti-rabbit IgG H&L (1:500, Jackson ImmunoResearch). The remaining slices were incubated with secondary antibodies for 2 hours at room temperature, according to the corresponding primary antibody combinations. The secondary antibodies were Cy™3-conjugated goat anti-rabbit IgG H&L (1:500, Jackson ImmunoResearch), Alexa Fluor® 488-conjugated guinea pig anti-mouse IgG H&L (1:500, Abcam), Alexa Fluor® 555-conjugated goat anti-mouse IgG H&L (1:500, Abcam), and Alexa Fluor® 488-conjugated goat anti-guinea pig IgG H&L (1:500, Abcam).

**Open field test (OFT)**

Mice were placed in the center of an arena (40 cm × 40 cm × 40 cm) for 5min freely moving. The movement of mice was tracked using the SMART 3.0 video tracking system (RWD, Shenzhen, China) positioned directly above the arena. The total distance and the mean velocity were analyzed to assess the motor function using the SMART 3.0 video analysis software

**HE staining**

The L4-L5 spinal cord segments and corresponding L5 DRG frozen sections underwent standard hematoxylin and eosin (HE) staining: immersion in Mayer's hematoxylin for nuclear staining (5 min), differentiation in acid alcohol, bluing in Scott's tap water, and counterstaining with eosin Y (1 min). Histological images were acquired using an inverted microscope (IX83; Olympus Corporation, Tokyo, Japan).

**Electroporation efficiency assessment**

MiR-101b-3p was labeled with Cy5 fluorescence (Sangon Biotech, China) for the electroporation efficiency analysis. Cy5 fluorescence detection was performed using a microplate reader (SyNERGY, BioTek, USA) at Cy5-miR-101b-3p concentrations of 1000, 500, 250, 125, 62.5, 31.25, 15.6, 7.8, 3.9, and 0 nM in 80 µL solution, as well as in NV samples both before and after electroporation. A standard curve was constructed by plotting the absorbance values (y-axis) against the corresponding gradient concentrations of Cy5-miR-101b-3p (x-axis). The concentration of miR-101b-3p in both NV and electroporated NV-miR samples was then calculated using the equation derived from the standard curve. Finally, the Cy5-miR-101b-3p level in NV before electroporation was compared with those in electroporated NV-miR.

**Flow cytometry**

Transfected cells expressing GFP fluorescence were incubated with 0.25% pancreatic enzyme (Gibco, USA) for 1-2 minutes to facilitate digestion. Neutralization was achieved by adding fetal bovine serum (FBS) and centrifugation at 250 × g for 3 minutes at room temperature. The resulting cell pellet was resuspended in 1 mL PBS, and the cell suspension was analyzed using a cytometer (BD Biosciences). Data was analyzed by FlowJo software version 10.8.1 (Becton, Dickinson and Company, USA).

**Local DRG injection**

According to the protocol, local DRG injection was performed as reported ^1^. 80 pmol siRNA was mixed with vivo-jetPEI® and glucose (at the ratio of N/P = 8, Polyplus Transferion® SA, France); and then incubated at room temperature for 15 minutes according to the protocol. DRG (right L4-L5) were surgically exposed and injected 1 μl siRNA mixture slowly by Hamilton syringe with a stereophonic positioning instrument (SYS-Micro4, WPI, Sarasota, FL, USA).

**Reference**

1. Wang H, Chen W, Dong Z, et al. A novel spinal neuron connection for heat sensation. *Neuron.* 2022;110(14):2315-2333.e2316.

**Supplementary figure legend**

**Supplementary Fig. 1. MSC viability by TNF-α** Cell viability of MSC after different concentrations of TNF-α stimulation (n = 5 dishes). MSC: Mesenchymal stem cell. All data are expressed as means ± SEM. ^##^ P < 0.01, ^###^ P < 0.001, ns: no significant.

**Supplementary Fig. 2** **Characteristics of C-EV and T-EV** (**A**) Morphology of MSC after TNFα stimulation (scale bar, 50 μm). (**B**) The expression of protein makers on C-EV and T-EV. (**C**) Mean diameter of C-EV and T-EV by NTA (n = 5 EV samples). (**D**) The concentration of EV (n = 5 EV samples). (**E**) The size distribution of C-EV. (**F**) The size distribution of T-EV. (**G**) PCA analysis of C-EV and T-EV (n = 3 EV samples). (**H**) Heatmap of the differential proteins between C-EV and T-EV. (**I**) Volcano of the differential proteins between C-EV and T-EV. C-BMSC: Bone marrow-derived MSC; T-BMSC: TNF-α-preconditioned bone marrow-derived MSC; C-EV: Control mesenchymal stem cell-derived extracellular vesicle; T-EV: TNF-α-preconditioned mesenchymal stem cell-derived extracellular vesicle. PCA: Principal Component Analysis. All data are expressed as median (IQR). ns: no significant.

**Supplementary Fig. 3 Distribution of EV in spinal dorsal horn and intermediolateral nucleus** (**A**) The distribution of PBS solution in SDH (scale bar, left, 200 μm, right, 20 μm). (**B**) The distribution of PKH-26-labeled C-EV in SDH. (**C**) The distribution of PKH-26-labeled T-EV in SDH. (**D**) The distribution of PBS solution in IML (scale bar, left, 200 μm; right, 20 μm). (**E**) The distribution of PKH-26-labeled C-EV in IML. (**F**) The distribution of PKH-26-labeled T-EV in IML. The white arrow indicates PKH-26-labeled EV; red color represents PKH-26-labeled EV, and green color indicates NeuN. EV: Extracellular vesicle. SDH: Spinal dorsal horn; IML: Intermediolateral nucleus.

**Supplementary Fig. 4 Different dosages of EV for analgesic effect.** (**A**) Paw withdrawal threshold after von Frey test following intrathecal delivery of 5 µg, 10 µg, and 20 µg of C-EV or T-EV (n = 5-6 mice per group). C-EV: Control mesenchymal stem cell-derived extracellular vesicle; T-EV: TNF-α-preconditioned mesenchymal stem cell-derived extracellular vesicle; CCI: chronic constriction injury. All data are expressed as means ± SEM. ^#^ P < 0.05, ^##^ P < 0.01, ^&^ P < 0.05, ^&&^ P < 0.01, ^*^P < 0.05, ns: no significant.

**Supplementary Fig. 5** **Behavioral, locomotor, and histological assessments following intrathecal administration of EV in *naïve* mice** (**A**) Paw withdrawal threshold after von-Frey test (n = 5 mice). (**B**) Brush response test (n = 5 mice). (**C**) Response time of pinprick test (n = 5 mice). (**D**) Latency to withdrawal after Hargreaves test (n = 5 mice). (**E**) Latency to withdrawal after ice-cold stimulation (n = 5 mice per group). (**F**) Total distance of mice before and after intrathecally delivering C-EV or T-EV (n = 5 mice). (**G**) Velocity of mice before and after intrathecally delivering C-EV or T-EV. (**H**) HE staining of DRG after intrathecal delivery of EV (scale bar, 50 μm). (I) HE staining of the spinal cord after intrathecal delivery of EV (scale bar, left, 200 μm; right, 100 μm). C-EV: Control mesenchymal stem cell-derived extracellular vesicle; T-EV: TNF-α-preconditioned mesenchymal stem cell-derived extracellular vesicle; HE: Hematoxylin-eosin. All data are expressed as means ± SEM. ns: no significant.

**Supplementary Fig. 6 Protein expression of inflammatory mediators by ELISA.** (**A**) Expression of IL-6 protein in DRG on Day 6 (n = 4 mice). (**B**) Expression of IL-10 protein in DRG on Day 6 (n = 3-4 mice). (**C**) Expression of IL-10 protein in DRG on Day 6 (n = 4 mice). All data are expressed as means ± SEM. ^#^ P < 0.01, ^##^ P < 0.01, ^###^ P < 0.001, ^&^ P < 0.05, ^&&&^ P < 0.001, ^**^ P < 0.01, ^***^ P < 0.001, ns: no significant.

**Supplementary Fig. 7 T-EV did not affect sciatic nerve conduction velocity** (A) Diagram of the experiment process for recording SEP. (B) Representative samples of somatosensory evoked potential across groups. The black arrow indicates the stimulus artifact. (C) Latency recording of CCI with stimulated ipsilesional sciatic nerves. (D) Conduction velocity recording of CCI with stimulated ipsilesional sciatic nerves. SEP: Somatosensory cortical evoked potential; CCI: Chronic constriction injury. ^#^ P < 0.05, ^##^ P < 0.01, ns: no significant.

**Supplementary Fig. 8 Differential miRNAs by RNA-seq** All differential miRNAs between C-EV and T-EV in a heatmap. C-EV: Control mesenchymal stem cell-derived extracellular vesicle; T-EV: TNF-α-preconditioned mesenchymal stem cell-derived extracellular vesicle.

**Supplementary Fig. 9** **TNF-α enhanced expression of miR-101b-3p in hBMSC-EV** (**A**) Diagram of experiment process. (**B**) miR-101b-3p expression in hBMSC-C-EV and hBMSC-T-EV (n = 5 EV samples). ^*^ P < 0.05, ns: no significant.

**Supplementary Fig. 10** **NF-κB inhibitor inhibited the expression of miR-101b-3p in T-EV**(**A**) Different doses of IN-11 were used to assess the inhibition rate on MSC. (**B**) Expression of miR-101b-3p in EV after IN-11 treatment (n = 3-4 EV samples). ^#^ P < 0.05, ^*^ P < 0.05, ^***^ P < 0.001, ns: no significant.

**Supplementary Fig. 11 Proteomic analysis of DRG** (**A**) Heatmap and function of differential proteins after DRG protein sequencing (n = 3 DRG). (**B**) GO analysis of differential proteins. DRG: dorsal root ganglion; GO: Gene ontology.

**Supplementary Fig. 12 Effect of miR-101b-3p on the expression of Nav1.7 and Nav1.8 on DRG** (**A**)The expression of Nav1.7 on DRG after intrathecal delivery of miR-101b-3p agomir by immunofluorescence (scale bar, left, 100 μm; right, 50μm). Red color indicates Nav1.7 expression, and green color indicates NeuN expression. (**B**) Semiquantitative analysis of DRG by immunofluorescence for Nav1.7 (n = 8-9 slices from 3 mice). (**C**) The expression of Nav1.8 on DRG after intrathecal delivery of miR-101b-3p agomir by immunofluorescence (scale bar, left, 100 μm; right, 50 μm). Red color indicates Nav1.8 expression, and green color indicates NeuN expression. (**D**) Semiquantitative analysis of DRG by immunofluorescence for Nav1.8 (n = 6-7 slices from 3 mice). CCI: chronic constriction injury. All data are expressed as means ± SEM. ns: no significant.

**Supplementary Fig. 13 The effect of EV on Nav1.6 expression of DRG neurons** (**A**) Fluorescence image showing the expression of Nav1.6 on DRG primary neurons from the CCI model following EV incubation (scale bar, 100 μm). Red color indicates Nav1.6 expression, and green color indicates NeuN expression. (**B**) Semiquantitative analysis of Nav1.6 expression by immunofluorescence for Nav1.6 (n = 12 cells). CCI: Chronic constriction injury. C-EV: Control extracellular vesicle; T-EV: TNF-α-preconditioned mesenchymal stem cell-derived extracellular vesicle; CCI: chronic constriction injury. All data are expressed as means ± SEM. ^*^ P < 0.05, ^###^ P < 0.001. ns: no significant.

**Supplementary Fig. 14 Silencing Nav1.6 has an analgesic effect** (**A**) Nav1.6 is expressed in IB4^+^, NF200^+,^ and CGRP^+^ neurons (scale bar, left, 100 μm, right, 50 μm). (**B**) The expression percentage of Nav1.6 on IB4^+^, CGRP^+^, and NF200^+^ neurons (n = 4-5 slices from 4-5 mice). (**C**) Schematic of siRNA delivery to DRG. (**D**) Fluorescence imaging of DRG after siRNA injection (scale bar, 100 μm). (**E**) Scn8a mRNA expression in DRG after siRNA injection by RT-qPCR (n = 4 DRG samples in sham group, n = 3 DRG samples in CCI group). (**F**) Von-Frey test after knockdown of Nav1.6 (n = 4 mice in sham group, n = 5 mice in CCI group). CCI: chronic constriction injury. All data are expressed as means ± SEM. ^*^ P < 0.05, ^**^ P < 0.01, ns: no significant.

**Supplementary Fig. 15** **Cy5-miR-101b-3p concentration in NV by electroporation** (**A**) Standard curve of Cy5-miR-101b-3p concentration and fluorescence intensity. (**B**) The Cy5-miR-101b-3p concentration in NV before and after electroporation. Cy5-miR-101b-3p: Cy5-labeled miR-101b-3p. ^**^ P < 0.01.

**Supplementary Fig. 16** **Characteristics of NV and NV-miR** (**A**) Mean size diameter of NV and NV-miR. (**B**) Size distribution of NV and NV-miR (n = 3 NV samples). NV: nanovesicle; NV-miR: NV-encapsulated transfected miR-101b-3p. All data are expressed as means (IQR). ns: no significant.

**Supplementary Fig. 17** **Behavioral, locomotor, and histological assessments following intrathecal administration of NV in *naïve* mice** (**A**) Paw withdrawal threshold after von-Frey test (n = 5 mice). (**B**) Brush response test (n = 5 mice). (**C**) Response time of the pinprick test (n = 5 mice). (**D**) Latency to withdrawal after Hargreaves test (n = 5 mice). (**E**) Latency to withdrawal after dry ice-cold stimulation (n = 5 mice). (**F**) Total distance of mice before and after intrathecally delivering NV or NV-miR (n = 5 mice). (**G**) Motor velocity of mice before and after intrathecally delivering NV or NV-miR. (**H**) HE staining of DRG after intrathecal delivery of NV and NV-miR (scale bar, 50 μm). (I) HE staining of the spinal cord after intrathecal delivery of NV and NV-miR (scale bar, left, 100 μm; right, 50 μm). HE: Hematoxylin-eosin. NV: nanovesicle; NV-miR: NV-encapsulated transfected miR-101b-3p. All data are expressed as means ± SEM. ns: no significant.

**Supplementary Fig. 18 *In vivo* tracing of NV and NV-miR** (**A**) DiR-labeled NV and NV-miR tracing by IVIS system (scale bar = 5 mm). (**B**) The maximum radiant efficiency of intrathecal DiR-NV and DiR-NV-miR. (**C**) Maximum diffusion distance of DiR-labeled NV or NV-miR from the injection site on Day 2. NV: nanovesicle; NV-miR: NV-encapsulated transfected miR-101b-3p; IVIS: *in vivo* tracing. ns: no significant.

**Supplementary Figure 19. Overview of the main experimental procedures** (**A**) Experimental design and workflow for Figure 1C-H. (**B**) Experimental design and workflow for Figure 2B-R. (**C**) Experimental design and workflow for Figures 3E-O and 6A-B. (**D**) Experimental design and workflow for Figure 4F-P. (**E**) Experimental design and workflow for Figure 5E-J. (**F**) Experimental design and workflow for Figure 7D-H. (**G**) Experimental design and workflow for Supplementary Figure 4. (**H**) Experimental design and workflow for Supplementary Figures 5A-G and 17A-G. (**I**) Experimental design and workflow for Supplementary Figures 14C-F and 18A-C. CCI: Chronic constriction injury; IVIS: In Vivo Imaging System; T-EV: TNF-α-preconditioned mesenchymal stem cell-derived extracellular vesicle; C-EV: Control mesenchymal stem cell-derived extracellular vesicle; IHC: Immunohistochemistry; NV: Nanovesicle; OFT: Open field test; NC: Negative control.

**Supplementary Table 1**

| Biologic Regent Name | |  | | | | |  | | | | | | | |  |
| --- | --- | --- | --- | --- | --- | --- | --- | --- | --- | --- | --- | --- | --- | --- | --- |
| Antibody | | **Company** | | |  | | **ID** | | |  |  |  |  |  |  |
| CD63 | | Abcam | | | | | AB216130 | | | | | | | |  |
| CD81 | | Santa Cruz | | | | | sc-166029 | | | | | | | |  |
| ALIX | | Proteintech | | | | | Cat No.12422-1-AP | | | | | | | |  |
| Phalloidin | | Solarbio | | | | | CA1620 | | | | | | | |  |
| Nav1.6 | | Abcam | | | | | AB302786 | | | | | | | |  |
| Nav1.7 | | Bioss | | | | | bs-21384R | | | | | | | |  |
| Nav1.8 | | Abcam | | | | | AB93616 | | | | | | | |  |
| NeuN | | Abcam | | | | | AB104224 | | | | | | | |  |
| NeuN | | Oasis biofarm | | | | | OB-PGP006 | | | | | | | |  |
| MAP2 | | Proteintech | | | | | Cat No. 67015-1-Ig | | | | | | | |  |
| CGRP | | Abcam | | | | | AB36001 | | | | | | | |  |
| IB4-FITC conjugated 488 | | Life | | | | | I21411 | | | | | | | |  |
| NF200 | | Millipore | | | | | MAB5262 | | | | | | | |  |
| Cyanine Cy^™^3 goat anti-rabbit IgG H&L | | Jackson ImmunoResearch | | | | | 111-165-003  RRID: AB_2338000 | | | | | | | |  |
| Alexa Fluor^®^ 488 guinea pig anti-mouse IgG H&L | | Abcam | | | | | AB150113 | | | | | | | |  |
| Alexa Fluor^®^ 555 goat anti-mouse IgG H&L | | Abcam | | | | | AB150114 | | | | | | | |  |
| Alexa Fluor^®^ 488 goat anti-guinea pig IgG H&L | | Abcam | | | | | AB150185 | | | | | | | |  |
| Regent Name | | **Resource** | | | | | **ID** | | | | | | | |  |
| Primary bone marrow-derived mesenchymal stem cell | | Cyagen Biosciences | | | | | MUBMX-01001 | | | | | | | |  |
| Dual-Luciferase Reporter Assay System | | Vazyme | | | | | DL101-01 | | | | | | | |  |
| In vivo-jetPEI^®^ | | Polyplus Transferion^®^SA | | | | | #101000030 | | | | | | | |  |
| Eastep® super total RNA extraction kit | | Promega | | | | | LS1040 | | | | | | | |  |
| SYBR master mix | | Vazyme | | | | | Q712-02 | | | | | | | |  |
| Lipofectamine™ 3000 transfection reagent | | Invitrogen™ | | | | | P5667270 | | | | | | | |  |
| TNF-α ELISA kit | | Ruixinbio | | | | | RX202412M | | | | | | | |  |
| IL-1β ELISA kit | | Ruixinbio | | | | | RX203063M | | | | | | | |  |
| IL-6 ELISA kit | | Ruixinbio | | | | | RX203049M | | | | | | | |  |
| IL-10 ELISA kit | | Ruixinbio | | | | | RX203075M | | | | | | | |  |
| CGRP ELISA kit | | Ruixinbio | | | | | RX202837M | | | | | | | |  |
| NF-κB-IN-11 | | MCE | | | | | Cat. No.HY-155998 | | | | | | | |  |
| Virus | | | | **Resource** | | | | | **Concentration** | | | | | |  |
| VSVG-LENTAI-hU6-shRNA(miR-101b-3p)-esEF1A-MataGFP-IRES-PuroR-WPRE-pA | | | | Taitool Bioscience | | | | | 1.01E+09 TU/mL | | | | | |  |
| VSVG-LENTAI-hU6-shRNA(NC)-esEF1A-MataGFP-IRES-PuroR-WPRE-pA | | | | Taitool Bioscience | | | | | 1.57E+09 TU/mL | | | | | |  |
| pDual-PGK-Fluc-Nav1.6 3’UTR-Mutated-pA-SV40-hRluc-Neo-pA | | | | Taitool Bioscience | | | | | 236 ng/μl | | | | | |  |
| pDual-PGK-Fluc-Nav1.6 3’UTR-pA-SV40-hRluc-Neo-pA12-(3,4) | | | | Taitool Bioscience | | | | | 276 ng/μl | | | | | |  |
| pAAV2-CAG-MasterRNAi155(miR-101b-3p)-EGFP-WPRE-pA | | | | Taitool Bioscience | | | | | 176 ng/μl | | | | | |  |
| pAAV2-CAG-MasterRNAi155(NC)-EGFP-WPRE-pA | | | | Taitool Bioscience | | | | | 627 ng/μl | | | | | |  |
| Gene name | | | **Sequence** | | | | | | | | | | | |  |
| miR-101b-3p | | | 5’-GUACAGUACUGUGAUAGCU-3’ | | | | | | | | | | | |  |
| let-7i-5p | | | 5’-UGAGGUAGUAGUUUGUGCUGUU-3’ | | | | | | | | | | | |  |
| miR-451a | | | 5’-AAACCGUUACCAUUACUGAGUU-3’ | | | | | | | | | | | |  |
| miRNA NC | | | 5’-UUGUACUACACAAAAGUACUG-3’ | | | | | | | | | | | |  |
| Nav1.6 siRNA | | | 5’-UAUACUGUUGAUUCUGUCCTT-3’ | | | | | | | | | | | |  |
| siRNA NC | | | 5’-ACGUGACACGUUCGGAGAA-3’ | | | | | | | | | | | |  |
| Oligonucleotides | **Forward primer** | | | | | **Reverse primer** | | | | | | | | |  |
| Scn8a | GGTGTTCTGCCTGAGTGTCTTCG | | | | | | | AGCCTCTGGTGCCGTTCTCC | | | | | | |  |
| Scn9a | CTTGTGTGCCTTATATTCTGGC | | | | | | | AACGGTTTGCAACTTGAGATAC | | | | | | |  |
| Scn10a | CAACAATTGCAGAAATCGAAGC | | | | | | | GTCTTCTCTCATTGGCGTTTTT | | | | | | |  |
| β-actin | CTACCTCATGAAGATCCTGACC | | | | | | | CACAGCTTCTCTTTGATGTCAC | | | | | | |  |
| Probe name | | **Sequence** | | | | | | | | | | | |  | |
| JWFFXTY-R | | GTGCAGGGTCCGAGGT | | | | | | | | | | | |  | |
| JWFTY-PROBE | | CAGAGCCACCTGGGCAATTT | | | | | | | | | | | |  | |
| U6-RT | | AACGCTTCACGAATTTGCGT | | | | | | | | | | | |  | |
| U6-S | | CTCGCTTCGGCAGCACA | | | | | | | | | | | |  | |
| U6-A | | AACGCTTCACGAATTTGCGT | | | | | | | | | | | |  | |
| U6 probe | | AGAAGATTAGCATGGCCCCTGCGCA | | | | | | | | | | | |  | |
| miR-101b-3p -RT | | CAGTGCAGGGTCCGAGGTCAGAGCCACCTGGGCAATTTTTTTTTTTAGCTAT | | | | | | | | | | | |  | |
| miR-101b-3p -F | | GGGCTACTGTGATAGCTAAAA | | | | | | | | | | | |  | |
| let-7i-5p-RT | | CAGTGCAGGGTCCGAGGTCAGAGCCACCTGGGCAATTTTTTTTTTTAACAGC | | | | | | | | | | | |  | |
| let-7i-5p-F | | CACGACCTGAGGTAGTAGTTTGT | | | | | | | | | | | |  | |
| miR-148a-3p -RT | | CAGTGCAGGGTCCGAGGTCAGAGCCACCTGGGCAATTTTTTTTTTTACAAAG | | | | | | | | | | | |  | |
| miR-148a-3p -F | | CCACCAAGCACTACAGAACTTTG | | | | | | | | | | | |  | |
| miR-143-3p -RT | | CAGTGCAGGGTCCGAGGTCAGAGCCACCTGGGCAATTTTTTTTTTTGAGCTA | | | | | | | | | | | |  | |
| miR-143-3p -F | | CCGTCCTGAGATGAAGCACT | | | | | | | | | | | |  | |
| miR-451a -RT | | CAGTGCAGGGTCCGAGGTCAGAGCCACCTGGGCAATTTTTTTTTTTAACTGA | | | | | | | | | | | |  | |
| miR-451a -F | | AACCACCAAACCGTTACCATTAC | | | | | | | | | | | |  | |
| miR-7a-5p-RT | | CAGTGCAGGGTCCGAGGTCAGAGCCACCTGGGCAATTTTTTTTTTTACAACA | | | | | | | | | | | |  | |
| miR-7a-5p-F | | CAGGTCGTGGAAGACTAGTGATTT | | | | | | | | | | | |  | |
| miR-218-5p -RT | | CAGTGCAGGGTCCGAGGTCAGAGCCACCTGGGCAATTTTTTTTTTTACATGG | | | | | | | | | | | |  | |
| miR-218-5p -F | | CAACCACCTTGTGCTTGATCTAA | | | | | | | | | | | |  | |
| miR-532-5p-RT | | CAGTGCAGGGTCCGAGGTCAGAGCCACCTGGGCAATTTTTTTTTTTACGGTC | | | | | | | | | | | |  | |
| miR-532-5p-F | | TCACCACATGCCTTGAGTGTAG | | | | | | | | | | | |  | |
| miR-206-3p-RT | | CAGTGCAGGGTCCGAGGTCAGAGCCACCTGGGCAATTTTTTTTTTTCCACAC | | | | | | | | | | | |  | |
| miR-206-3p-F | | CACAGGAGTGGAATGTAAGGAAGT | | | | | | | | | | | |  | |
